# Supplementary figures and images for: Structural characterization and evaluation of antimicrobial and cytotoxic activity of six plant phenolic acids
Source: PLoS One. 2024 Jun 17;19(6):e0299372. doi: 10.1371/journal.pone.0299372 (PMC11182523; doi:10.1371/journal.pone.0299372)

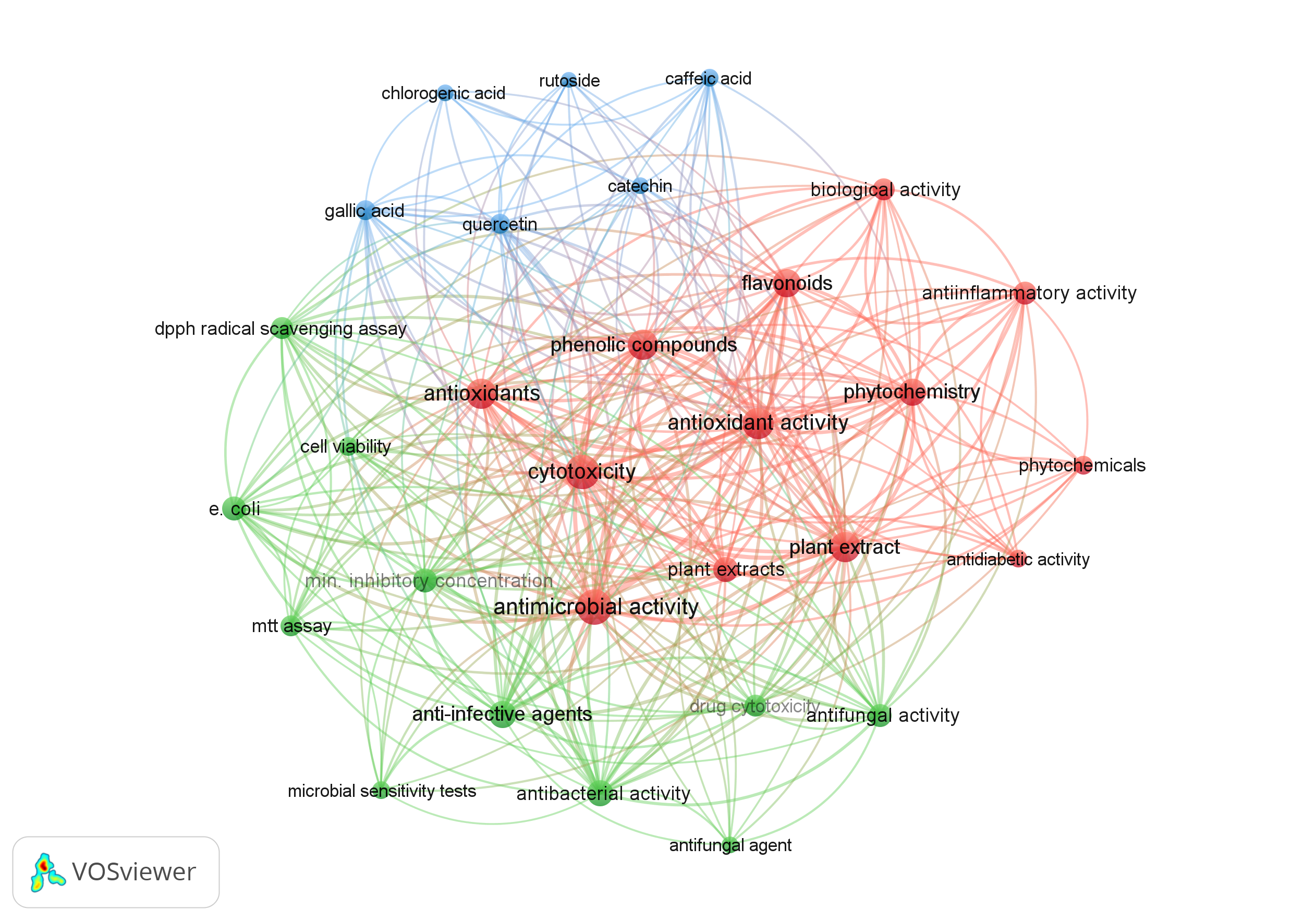

Supplement: S1 Fig — (TIFF) [file pone.0299372.s001.tiff]

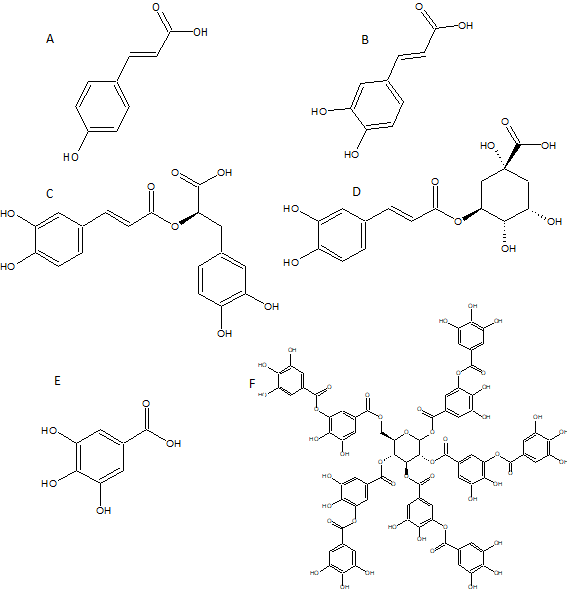

Supplement: S2 Fig — Structures of p-coumaric acid (A), caffeic acid (B), rosmarinic acid (C), chlorogenic acid (D), gallic acid (E) and tannic acid (F). (TIF) [file pone.0299372.s002.tif]
