# Supplementary material for: Structural characterization and evaluation of antimicrobial and cytotoxic activity of six plant phenolic acids
Source: PLoS One. 2024 Jun 17;19(6):e0299372. doi: 10.1371/journal.pone.0299372 (PMC11182523; doi:10.1371/journal.pone.0299372)
Supplement: S1 Table — (PDF) [file pone.0299372.s003.pdf]

Compound	Value	Unit	Standard	Microorganism strain	Reference
GA	17.6	mM	MIC*	*E. coli*	[1]
GA	35.3	mM	MIC*	*S. aureus*	[1]
GA	17.6	mM	MIC*	*B. subtilis*	[1]
GA	17.6	mM	MIC*	*S. enteritidis*	[1]
GA	17.6	mM	MIC*	*C. albicans*	[1]
GA	11.0 ± 0.35	mM	IZ	*P. fluorescens*	[2]
GA	13.2 ± 0.19	mM	IZ	*P30-4*	[2]
GA	15.0 ± 0.9	mM	IZ	*P. fragi*	[2]
GA	14.8 ± 0.4	mM	IZ	*P.putida*	[2]
GA	29.4	mM	MIC	*P. fluorescens*	[2]
GA	29.4	mM	MIC	*P30-4*	[2]
GA	14.7	mM	MIC	*P. fragi*	[2]
GA	14.7	mM	MIC	*P. putida*	[2]
GA	0.37	mM	MIC	S. mutans	[3]
GA	0.59	mM	MIC	*P. aeruginosa*	[3]
GA	1.47	mM	MIC	*M. haemolytica*	[4]
GA	2.93	mM	MIC	*P. multocida*	[4]
5-CQA	28.22	mM	MIC	*Staphylococcus aureus*	[5]
5-CQA	28.22	mM	MIC	*Enterococcus faecium*	[5]
5-CQA	28.22	mM	MIC	*Escherichia coli*	[5]
5-CQA	28.22	mM	MIC	*Proteus vulgaris*	[5]
5-CQA	28.22	mM	MIC	*Pseudomonas aeruginosa*	[5]
5-CQA	14.11	mM	MIC	*Klebsiella pneumoniae*	[5]
5-CQA	28.22	mM	MIC	*Candida albicans*	[5]
5-CQA	19.93	mM	MIC	*E. coli*	[6]
5-CQA	>19.93	mM	MIC	Bacillus sp.	[6]
5-CQA	>19.93	mM	MIC	Staphylococcus Epidermidis	[6]
5-CQA	>19.93	mM	MIC	*Streptococcus Pyogenes*	[6]
5-CQA	>39.82	mM	MIC	Candida sp.	[6]
5-CQA	14.5+ 0.50	mM	IZ	*Alicyclobacillus acidoterrestris*	[7]
5-CQA	0.006	mM	MIC	*Alicyclobacillus acidoterrestris*	[7]
5-CQA	11.29	mM	MBC	*Alicyclobacillus acidoterrestris*	[7]
5-CQA	0.23	mM	MIC	*Candida albicans ATCC 90028*	[8]
5-CQA	100	%	Inhibitory activity for C=5 [mM]	*Staphylococcus aureus CCM 4516*	[9]
5-CQA	100	%	Inhibitory activity for C=5 [mM]	*Pseudomonas aeruginosa CCM 1961*	[9]

	100			*Escherichia coli CCM 4517*	
	100			*Candida albicans CCM 8215*	
	100			*Aspergillus brasiliensis CCM 8222*	
**CA**	1.39	mM	MIC	*E. coli*	[10]
	>2.78			*Bacillus sp.*	
	>2.78			*Staphylococcus epidermidis*	
	>2.78			*Streptococcus pyogenes*	
	>5.55			*Candida sp.*	
	1.42	mM	MIC	*S. aureus ATCC 25923*	[11]
	1.42			*S. aureus ATCC 43300*	
	1.42			*S. aureus ATCC 6538*	
	100	%	Inhibitory activity for C = 5 [mM]	*Staphylococcus aureus CCM 4516*	[9]
	50-99			*Pseudomonas aeruginosa CCM 1961*	
	100			*Escherichia coli CCM 4517*	
	100			*Candida albicans CCM 8215*	
	100			*Aspergillus brasiliensis CCM 8222*	
	72.16	mM	MIC	*Escherichia coli*	[12]
	99.91			*Bacillus subtilis*	
	160.97			*Candida albicans*	
	5.68	mM	MIC	*S. aureus*	[13]
**p-CA**	0.25	mM	MIC	*Bacillus cereus*	[14]
	0.63			*S. typhimurium*	
	-	mM	IZ	*Pseudomonas aeruginosa*	[15]
	-			*Escherichia coli*	
	9±0.6			*Bacillus cereus*	
	-			*Staphylococcus aureus*	
	-			*Aspergillus Niger*	
	-			*Candida albicans*	
	1.22	mM	MIC	*Alicyclobacillus acidoterrestris*	[16]
	>9.75		MBC		
	30.48	mM	MIC	*C. sakazakii N112*	[17]
	15.24			*C. sakazakii N13*	
	15.24			*C. sakazakii ATCC*	
	3.66	mM	MIC	*Staphylococcus aureus*	[18]
**RA**	0.69	mM	MIC	*E. coli*	[10]
	>1.39			*Bacillus sp.*	

	>1.39			*Staphylococcus epidermidis*	
	>1.39			*Streptococcus pyogenes*	
	11.6±0.4	mM	IZ C = 2 [mg/ml]	*Methicillin-resistant Staphylococcus aureus*	[19]
	27.75	mM	MIC	*Methicillin-resistant Staphylococcus aureus*	
	12.4±0.4	mM	IZ C = 0.2 [mg/ml]	*S. aureus*	
	2.22	mM	MIC	*S. aureus*	
	3.47	mM	MIC	*S. aureus CCM 4750*	[20]
	3.47			*E. faecalis CCM 4224*	
	3.47			*P. aeruginosa CCM 3955*	
	3.47			*K.pneumoniae CCM 4415*	
	1.73			*E. coli CCM 3954*	
	3.47			*P. mirabilis CCM 7188*	
	0.5			*Trichoderma viridae*	
	>0.5			*Aspergillus flavus*	
	>0.5			*Aspergillus Niger*	
	0.5			*Mucor racemosus*	
	1.73			*Candida albicans CCM 90028*	
**TA**	-	mM	MIC	*E. coli*	[21]
	0.04			*P. aeruginosa*	
	0.04			*B. subtilis*	
	0.02			*S. aureus*	
	0.04			*C. albicans*	
	13.33 ± 0.57	mM	IZ	*E.coli*	[22]
	10.16 ± 1.04			*S. aureus*	
	30	mM	IZ	*E.coli*	[23]
	32			*S. aureus*	
	0.24	mM	MQIC	*S.Typhi*	[24]
	-	-	IZ	*S.Paratyphi*	
	-	-	IZ	*S.Typhi*	
	-	-	IZ	*S.Paratyphi*	
	0.02-0.09	mM	MIC	*Staphylococcus aureus*	[25]
	0.04	mM	MIC	MRSA	[26]

[1] M. Kalinowska *et al.*, "Plant-Derived and Dietary Hydroxybenzoic Acids—A Comprehensive Study of Structural, Anti-/Pro-Oxidant, Lipophilic, Antimicrobial, and Cytotoxic Activity in

MDA-MB-231 and MCF-7 Cell Lines," *Nutrients*, vol. 13, no. 9, p. 3107, Sep. 2021, doi: 10.3390/nu13093107.

[2] E. Sorrentino *et al.*, "Antimicrobial activity of gallic acid against food-related Pseudomonas strains and its use as biocontrol tool to improve the shelf life of fresh black truffles," *Int J Food Microbiol*, vol. 266, pp. 183–189, Feb. 2018, doi: 10.1016/j.ijfoodmicro.2017.11.026.

[3] P. M. Osamudiamen, B. B. Oluremi, O. O. Oderinlo, and O. O. Aiyelaagbe, "Trans-resveratrol, piceatannol and gallic acid: Potent polyphenols isolated from Mezoneuron benthamianum effective as anticaries, antioxidant and cytotoxic agents," *Sci Afr*, vol. 7, p. e00244, Mar. 2020, doi: 10.1016/J.SCIAF.2019.E00244.

[4] K. Rajamanickam, J. Yang, and M. K. Sakharkar, "Gallic Acid Potentiates the Antimicrobial Activity of Tulathromycin Against Two Key Bovine Respiratory Disease (BRD) Causing-Pathogens," *Front Pharmacol*, vol. 9, Jan. 2019, doi: 10.3389/fphar.2018.01486.

[5] E. Bajko, M. Kalinowska, P. Borowski, L. Siergiejczyk, and W. Lewandowski, "5-O-Caffeoylquinic acid: A spectroscopic study and biological screening for antimicrobial activity," *LWT - Food Science and Technology*, vol. 65, pp. 471–479, Jan. 2016, doi: 10.1016/j.lwt.2015.08.024.

[6] M. Kalinowska, E. Bajko, M. Matejczyk, P. Kaczyński, B. Łozowicka, and W. Lewandowski, "The Study of Anti-/Pro-Oxidant, Lipophilic, Microbial and Spectroscopic Properties of New Alkali Metal Salts of 5-O-Caffeoylquinic Acid," *Int J Mol Sci*, vol. 19, no. 2, p. 463, Feb. 2018, doi: 10.3390/ijms19020463.

[7] R. Cai *et al.*, "Antibacterial activity and mechanism of cinnamic acid and chlorogenic acid against *Alicyclobacillus acidoterrestris* vegetative cells in apple juice," *Int J Food Sci Technol*, vol. 54, no. 5, pp. 1697–1705, May 2019, doi: 10.1111/ijfs.14051.

[8] J. Yun and D. G. Lee, "Role of potassium channels in chlorogenic acid-induced apoptotic volume decrease and cell cycle arrest in Candida albicans," *Biochimica et Biophysica Acta (BBA) - General Subjects*, vol. 1861, no. 3, pp. 585–592, Mar. 2017, doi: 10.1016/j.bbagen.2016.12.026.

[9] J. Kyselka *et al.*, "Antioxidant and antimicrobial activity of linseed lignans and phenolic acids," *European Food Research and Technology*, vol. 243, no. 9, pp. 1633–1644, Sep. 2017, doi: 10.1007/s00217-017-2871-9.

[10] M. Matejczyk, R. Świsłocka, A. Golonko, W. Lewandowski, and E. Hawrylik, "Cytotoxic, genotoxic and antimicrobial activity of caffeic and rosmarinic acids and their lithium, sodium and potassium salts as potential anticancer compounds," *Adv Med Sci*, vol. 63, no. 1, pp. 14–21, Mar. 2018, doi: 10.1016/j.advms.2017.07.003.

[11] M. Kępa *et al.*, "Antimicrobial Potential of Caffeic Acid against *Staphylococcus aureus* Clinical Strains," *Biomed Res Int*, vol. 2018, pp. 1–9, Jul. 2018, doi: 10.1155/2018/7413504.

[12] Ż. Arciszewska *et al.*, "Caffeic Acid/Eu(III) Complexes: Solution Equilibrium Studies, Structure Characterization and Biological Activity," *Int J Mol Sci*, vol. 23, no. 2, p. 888, Jan. 2022, doi: 10.3390/ijms23020888.

[13] J. F. S. dos Santos *et al.*, "In vitro e in silico evaluation of the inhibition of Staphylococcus aureus efflux pumps by caffeic and gallic acid," *Comp Immunol Microbiol Infect Dis*, vol. 57, pp. 22–28, Apr. 2018, doi: 10.1016/j.cimid.2018.03.001.

[14] A. Bag and R. R. Chattopadhyay, "Synergistic antibacterial and antibiofilm efficacy of nisin in combination with *p* -coumaric acid against food-borne bacteria *Bacillus cereus* and *Salmonella typhimurium*," *Lett Appl Microbiol*, vol. 65, no. 5, pp. 366–372, Nov. 2017, doi: 10.1111/lam.12793.

[15] M. Abdel-Wareth and M. Ghareeb, "Bioprospecting certain freshwater-derived fungi for phenolic compounds with special emphasis on antimicrobial and larvicidal activity of methyl gallate and p-coumaric Acid," *Egypt J Chem*, vol. 0, no. 0, pp. 0–0, Jun. 2018, doi: 10.21608/ejchem.2018.3237.1276.

[16] J. Li *et al.*, "Deciphering the antibacterial activity and mechanism of p-coumaric acid against Alicyclobacillus acidoterrestris and its application in apple juice," *Int J Food Microbiol*, vol. 378, p. 109822, Oct. 2022, doi: 10.1016/j.ijfoodmicro.2022.109822.

[17] R. Chauhan, W. Azmi, S. Bansal, and G. Goel, "Multivariate analysis of adaptive response to ferulic acid and *p* -coumaric acid after physiological stresses in *Cronobacter sakazakii*," *J Appl Microbiol*, vol. 131, no. 6, pp. 3069–3080, Dec. 2021, doi: 10.1111/jam.15164.

[18] S. Cui *et al.*, "Phenolic acids derived from rice straw generate peroxides which reduce the viability of Staphylococcus aureus cells in biofilm," *Ind Crops Prod*, vol. 140, p. 111561, Nov. 2019, doi: 10.1016/j.indcrop.2019.111561.

[19] S. Ekambaram, S. Perumal, A. Balakrishnan, N. Marappan, S. Gajendran, and V. Viswanathan, "Antibacterial synergy between Rosmarinic acid and antibiotics against Methicillin resistant Staphylococcus aureus," *J Intercult Ethnopharmacol*, vol. 5, no. 4, p. 358, 2016, doi: 10.5455/jice.20160906035020.

[20] S. Bittner Fialová *et al.*, "Derivatization of Rosmarinic Acid Enhances its in vitro Antitumor, Antimicrobial and Antiprotozoal Properties," *Molecules*, vol. 24, no. 6, p. 1078, Mar. 2019, doi: 10.3390/molecules24061078.

[21] N. Sahiner, S. Sagbas, M. Sahiner, C. Silan, N. Aktas, and M. Turk, "Biocompatible and biodegradable poly(Tannic Acid) hydrogel with antimicrobial and antioxidant properties," *Int J Biol Macromol*, vol. 82, pp. 150–159, Jan. 2016, doi: 10.1016/j.ijbiomac.2015.10.057.

[22] H. Kim *et al.*, "Facile thermal and hydrolytic conversion of tannic acid: Enhancement of antimicrobial activity and biocompatibility for biomedical applications," *Mater Chem Phys*, vol. 285, p. 126141, Jun. 2022, doi: 10.1016/j.matchemphys.2022.126141.

[23] A. S. Sethulekshmi *et al.*, "Multifunctional role of tannic acid in improving the mechanical, thermal and antimicrobial properties of natural rubber-molybdenum disulfide nanocomposites," *Int J Biol Macromol*, vol. 225, pp. 351–360, Jan. 2023, doi: 10.1016/j.ijbiomac.2022.11.054.

[24] C. Sivasankar, N. K. Jha, R. Ghosh, and P. H. Shetty, "Anti quorum sensing and anti virulence activity of tannic acid and it's potential to breach resistance in Salmonella enterica Typhi / Paratyphi A clinical isolates," *Microb Pathog*, vol. 138, p. 103813, Jan. 2020, doi: 10.1016/j.micpath.2019.103813.

[25] W. Jing, C. Xiaolan, C. Yu, Q. Feng, and Y. Haifeng, "Pharmacological effects and mechanisms of tannic acid," *Biomedicine & Pharmacotherapy*, vol. 154, p. 113561, Oct. 2022, doi: 10.1016/j.biopha.2022.113561.

[26] G. Dong *et al.*, "Antimicrobial and anti-biofilm activity of tannic acid against *Staphylococcus aureus*," *Nat Prod Res*, vol. 32, no. 18, pp. 2225–2228, Sep. 2018, doi: 10.1080/14786419.2017.1366485.
