# Supplementary material for: Structural characterization and evaluation of antimicrobial and cytotoxic activity of six plant phenolic acids
Source: PLoS One. 2024 Jun 17;19(6):e0299372. doi: 10.1371/journal.pone.0299372 (PMC11182523; doi:10.1371/journal.pone.0299372)
Supplement: S2 Table — (PDF) [file pone.0299372.s004.pdf]

Compound	Value	Unit	Standard	Assay	Cell Line	Source
GA	50	µM	GI 50	MTT	A2780	[1]
	50				H460	
	50				A431	
	50				MCF-7	
	50				MCF10A	
	0.12	mM	IC 50	MTT	T47D	[2]
	29.92	%	Inhibition percentage	MTT within 4 h in GArGO (50 µg/ml) alone treated samples	IP15	[3]
	11.45	mM	IC 50	MTT	HeLa cells	[4]
	0.06	mM	IC 50	MTT	MRC-5	[5]
	0.26	mM	IC 50	MTT	MDA-MB-231	[6]
	0.11	mM	IC 50	MTT	MCF-7	[7]
	0.11	mM	IC_{50}	AlamarBlue and CCK08 assays	A2780S	[8]
	0.21				A2780CP	
	0.29				HOSE6-3	
	0.06	mM	IC 50	MTT	Caco-2	[9]
	304.35	mM	IC 50	Cell counting kit-8 (CCK-8) assay	A549	[10]
5-CQA	27.66	mM	IC 50	Cell counting kit-8 (CCK-8) assay	A549	[11]
	758 ± 19.09	µM	EC 50	LDH assay	Caco-2	[12]
	6.36	%	Kill %	PI assay for 200 µM	U2OS	[13]
	5.37				MG-63	
	71%	%	cell number reduction	MTT for 400 µM	Saos-2	[14]
	40				MG-63	
	74				U2OS	
	40	µM	IC 50	CCK-8 assay	A498	[15]
	-	-	IC 50		HEK293	
	15	%	apoptotic rate	Annexin V/PI dual staining assay for 20 µM	MDA-MB-23	[16]
	23				4T1	
	952 ± 32.5	µM	IC_{50}	iCELLigence System	MCF-7	[17]
	940 ± 21.2				SK-BR-3	
	590.5 ± 10.6				MDA-MB-231	
	882.5 ± 12.0				MDA-MB-468	

	1095 ± 121.6				BT-20	
CA	10	µM	IC_{50}	Antiproliferative activity against human cells by CellTiter-Glo assay	MV4-11	[18]
	10	µM	IC_{50}		MOLM-14	
	6.4	µM	IC_{50}		MOLM-13	
	49.4	mM	IC_{50}	Cell counting kit-8 (CCK-8) assay	A549 cells	[11]
	76	µM	IC_{50}	Neuroprotection against amyloid beta (25 to 35)-induced cell death in rat PC12 cells pre-incubated for 3 h followed by amyloid beta addition measured after 24 h by MTT assay	PC-12	[19]
	102.98	µM	IC_{50}	XTT -NR-SRB assay	MCF-7	[20]
	83.47			SRB assay		
	84.87			NR assay		
	1	mM	IC_{50}	MTT	U118MG	[21]
	4				DLD-1	
	826.59	µM	EC_{50}	MTT	HCT116	[22]
	1.355	mM	IC_{50}	MTT	HEp-2	[23]
	1.50	mM	IC_{50}	MTT	MCF7	[24]
	0.68				MDAMB-231	
	1.45				SK-BR-3	
	0.86				HeLa	
	0.88				HT-29	
	0.58				Neuro 2A	
	1.35				PC-3	
	0.60				Vero	
	460 ± 21.88	µM	EC_{50}	MTT	Caco-2	[12]
p-CA	4.88	mM	IC_{50}	MTT	A375	[25]
	6.10	mM			SK-MEL-37	
	603.4	µM	IC_{50}	MTT	786-O	[26]
	3.36	mM	IC_{50}	MTT	AGS	[27]
	1.39	mM	IC_{50}	MTT	MCF-7	[24]
	0.94				MDAMB-231	
	0.74				SK-BR-3	
	0.01				HeLa	
	0.15				HT-29	
	0.81				Neuro 2A	
	0.84				PC-3	
	0.38				Vero	
	223	µM	IC_{50}	Alamar Blue assay	DLD-1	[28]

	1.6	mM			Caco-2	
**RA**	2.9	µM	IC_{50}	Antiproliferative activity against human cells by CellTiter-Glo assay	MOLM-13	[18]
	10	µM	IC_{50}		MV4-11	
	7.1	µM	IC_{50}		MOLM-14	
	2.22	mM	IC_{50}	MTT	MCF-7	[24]
	0.34				MDAMB-231	
	0.41				SK-BR-3	
	0.39				HeLa	
	0.42				HT-29	
	0.47				Neuro 2A	
	1.07				PC-3	
	1.08				Vero	
	34.6	µM	IC_{50}	MTT	OCVAR-3	[29]
**TA**	4.2	µM	IC_{50}	MTT	Hs 683	[30]
	50	µM	IC_{50}	Crystal violet assay	A549	[31]
	2.5	µM	IC_{50}	MTT	MDA-MB-231	[32]
	4				MCF-7	
	46.29 ± 1.86	µM	IC_{50}	MTT	Caco-2	[33]
	53.6	µM	IC_{50}	MTT	DLD-1	[34]
	43.1	µM			HCT-116	
	50	µM	IC_{50}	MTT	FaDu	[35]
	13 ± 2	µM	IC_{50}	CellTiter 96® AQueous One Solution Cell Proliferation Assay kit	CaSki	[36]
	22 ± 3				HeLa	
	12.9 ± 1.8				PC3	
	23 ± 0.8				Hep G2	
	11 ± 1.2				Hep3B	
	24 ± 0.2				IHH	

[1] H. N. T. Pham, J. A. Sakoff, Q. Van Vuong, M. C. Bowyer, and C. J. Scarlett, “Comparative cytotoxic activity between kaempferol and gallic acid against various cancer cell lines,” *Data Brief*, vol. 21, pp. 1033–1036, Dec. 2018, doi: 10.1016/J.DIB.2018.10.121.

[2] N. M. Aborehab and N. Osama, “Effect of Gallic acid in potentiating chemotherapeutic effect of Paclitaxel in HeLa cervical cancer cells,” *Cancer Cell Int*, vol. 19, no. 1, 2019, doi: 10.1186/s12935-019-0868-0.

[3] W. Peng, P. Luo, D. Gui, W. Jiang, H. Wu, and J. Zhang, “Enhanced anticancer effect of fabricated gallic acid/CdS on the rGO nanosheets on human glomerular mesangial (IP15) and

epithelial proximal (HK2) kidney cell lines - Cytotoxicity investigations," *J Photochem Photobiol B*, vol. 178, pp. 243–248, Jan. 2018, doi: 10.1016/J.JPHOTOBIOL.2017.11.012.

[4] S. A. Yahya and F. A. Hassan, "Cytotoxic Effects Of Gallic Acid In Human Breast Cancer Cell Mda-Mb-231," *Journal of Pharmaceutical Negative Results ¦*, vol. 13, p. 2022, doi: 10.47750/pnr.2022.13.S08.166.

[5] N. Al Balushi *et al.*, "Addition of Gallic Acid Overcomes Resistance to Cisplatin in Ovarian Cancer Cell Lines," *Asian Pacific Journal of Cancer Prevention*, vol. 23, no. 8, 2022, doi: 10.31557/APJCP.2022.23.8.2661.

[6] S. A. Yahya and F. A. Hassan, "Cytotoxic Effects Of Gallic Acid In Human Breast Cancer Cell Mda-Mb-231," *J Pharm Negat Results*, vol. 13, pp. 1337–1344, Nov. 2022, doi: 10.47750/PNR.2022.13.S08.166.

[7] H. Rezaei-Seresht, H. Cheshomi, F. Falanji, F. Movahedi-Motlagh, M. Hashemian, and E. Mireskandari, "Cytotoxic activity of caffeic acid and gallic acid against MCF-7 human breast cancer cells: An in silico and in vitro study.," *Avicenna J Phytomed*, vol. 9, no. 6, pp. 574–586, 2019, doi: 10.22038/AJP.2019.13475.

[8] N. Al Balushi *et al.*, "Addition of Gallic Acid Overcomes Resistance to Cisplatin in Ovarian Cancer Cell Lines," *Asian Pacific Journal of Cancer Prevention*, vol. 23, no. 8, pp. 2661–2669, Aug. 2022, doi: 10.31557/APJCP.2022.23.8.2661.

[9] I. Y. M. Ho, A. Abdul Aziz, and S. Mat Junit, "Evaluation of Anti-proliferative Effects of Barringtonia racemosa and Gallic Acid on Caco-2 Cells," *Sci Rep*, vol. 10, no. 1, p. 9987, Jun. 2020, doi: 10.1038/s41598-020-66913-x.

[10] X. Bai, T. Lai, T. Zhou, Y. Li, X. Li, and H. Zhang, "In vitro antioxidant activities of phenols and oleanolic acid from mango peel and their cytotoxic effect on a549 cell line," *Molecules*, vol. 23, no. 6, 2018, doi: 10.3390/molecules23061395.

[11] X. Bai, T. Lai, T. Zhou, Y. Li, X. Li, and H. Zhang, "In Vitro Antioxidant Activities of Phenols and Oleanolic Acid from Mango Peel and Their Cytotoxic Effect on A549 Cell Line," *Molecules 2018, Vol. 23, Page 1395*, vol. 23, no. 6, p. 1395, Jun. 2018, doi: 10.3390/MOLECULES23061395.

[12] S. Sadeghi Ekbatan, X.-Q. Li, M. Ghorbani, B. Azadi, and S. Kubow, "Chlorogenic Acid and Its Microbial Metabolites Exert Anti-Proliferative Effects, S-Phase Cell-Cycle Arrest and Apoptosis in Human Colon Cancer Caco-2 Cells," *Int J Mol Sci*, vol. 19, no. 3, p. 723, Mar. 2018, doi: 10.3390/ijms19030723.

[13] A. Salzillo, A. Ragone, A. Spina, S. Naviglio, and L. Sapio, "Chlorogenic Acid Enhances Doxorubicin-Mediated Cytotoxic Effect in Osteosarcoma Cells," *Int J Mol Sci*, vol. 22, no. 16, p. 8586, Aug. 2021, doi: 10.3390/ijms22168586.

[14] L. Sapio *et al.*, "Chlorogenic acid activates ERK1/2 and inhibits proliferation of osteosarcoma cells," *J Cell Physiol*, vol. 235, no. 4, pp. 3741–3752, Apr. 2020, doi: 10.1002/jcp.29269.

[15] X. Wang *et al.*, "Chlorogenic acid inhibits proliferation and induces apoptosis in A498 human kidney cancer cells via inactivating PI3K/Akt/mTOR signalling pathway," *Journal of Pharmacy and Pharmacology*, vol. 71, no. 7, pp. 1100–1109, Jun. 2019, doi: 10.1111/jphp.13095.

[16] A. Zeng *et al.*, “Chlorogenic acid induces apoptosis, inhibits metastasis and improves antitumor immunity in breast cancer via the NF-κB signaling pathway,” *Oncol Rep*, vol. 45, no. 2, pp. 717–727, Dec. 2020, doi: 10.3892/or.2020.7891.

[17] A. Atalay and O. Bender, “Evaluation of anti-proliferative and cytotoxic effects of chlorogenic acid on breast cancer cell lines by real-time, label-free and high-throughput screening,” *Marmara Pharm J*, vol. 22, no. 2, pp. 173–179, Apr. 2018, doi: 10.12991/mpj.2018.54.

[18] Q. Wang *et al.*, “Salviachinensines A-F, Antiproliferative Phenolic Derivatives from the Chinese Medicinal Plant Salvia chinensis,” *J Nat Prod*, vol. 81, no. 11, pp. 2531–2538, Nov. 2018, doi: 10.1021/ACS.JNATPROD.8B00638/SUPPL_FILE/NP8B00638_SI_001.PDF.

[19] A. Iraji *et al.*, “Multifunctional iminochromene-2H-carboxamide derivatives containing different aminomethylene triazole with BACE1 inhibitory, neuroprotective and metal chelating properties targeting Alzheimer’s disease,” *Eur J Med Chem*, vol. 141, pp. 690–702, Dec. 2017, doi: 10.1016/J.EJMECH.2017.09.057.

[20] A. Kabała-Dzik, A. Rzepecka-Stojko, R. Kubina, R. D. Wojtyczka, E. Buszman, and J. Stojko, “Caffeic Acid Versus Caffeic Acid Phenethyl Ester in the Treatment of Breast Cancer MCF-7 Cells: Migration Rate Inhibition,” *Integr Cancer Ther*, vol. 17, no. 4, pp. 1247–1259, Dec. 2018, doi: 10.1177/1534735418801521.

[21] M. Naumowicz *et al.*, “The influence of the pH on the incorporation of caffeic acid into biomimetic membranes and cancer cells,” *Sci Rep*, vol. 12, no. 1, p. 3692, Mar. 2022, doi: 10.1038/s41598-022-07700-8.

[22] M. Secme, D. Mutlu, L. Elmas, and S. Arslan, “Assessing effects of caffeic acid on cytotoxicity, apoptosis, invasion, GST enzyme activity, oxidant, antioxidant status and micro-RNA expressions in HCT116 colorectal cancer cells,” *South African Journal of Botany*, vol. 157, pp. 19–26, Jun. 2023, doi: 10.1016/J.SAJB.2023.03.046.

[23] L. M. da Silva, Y. Frión-Herrera, A. R. Bartolomeu, C. M. Gorgulho, and J. M. Sforcin, “Mechanisms involved in the cytotoxic action of Brazilian propolis and caffeic acid against HEp-2 cells and modulation of P-glycoprotein activity,” *Journal of Pharmacy and Pharmacology*, vol. 69, no. 11, pp. 1625–1633, Oct. 2017, doi: 10.1111/jphp.12789.

[24] C. Sevimli-Gur and O. Yesil-Celiktas, “Cytotoxicity screening of supercritical fluid extracted seaweeds and phenylpropanoids,” *Mol Biol Rep*, vol. 46, no. 4, pp. 3691–3699, Aug. 2019, doi: 10.1007/s11033-019-04812-9.

[25] Z. Kianmehr, K. Khorsandi, M. Mohammadi, and R. Hosseinzadeh, “Low-level laser irradiation potentiates anticancer activity of p-coumaric acid against human malignant melanoma cells,” *Melanoma Res*, vol. 30, no. 2, pp. 136–146, Apr. 2020, doi: 10.1097/CMR.0000000000000603.

[26] R. Caparica, A. Rolim Baby, T. Almeida, and J. Guilherme Costa, “In vitro cytotoxicity assessment of ferulic, caffeic and p-coumaric acids on human renal cancer cells,” *Biomedical and Biopharmaceutical Research Journal*, vol. 17, no. 1, pp. 1–12, Mar. 2020, doi: 10.19277/bbr.17.1.225.

[27] Y. Sefidi-Heris, E. Zarei, and I. Saadat, “Metformin and p-coumaric acid downregulate the expression of hTERT in gastric cancer cell line AGS,” *Gene Rep*, vol. 32, 2023, doi: 10.1016/j.genrep.2023.101795.

[28] S. KARAKURT, G. ABUŞOĞLU, and Z. C. ARITULUK, “Comparison of anticarcinogenic properties of Viburnum opulus and its active compound p-coumaric acid on human colorectal

carcinoma," *TURKISH JOURNAL OF BIOLOGY*, vol. 44, no. 5, pp. 252–263, Oct. 2020, doi: 10.3906/biy-2002-30.

[29] Y. Zhang *et al.*, "Anticancer effects of Rosmarinic acid in OVCAR-3 ovarian cancer cells are mediated via induction of apoptosis, suppression of cell migration and modulation of lncRNA MALAT-1 expression.," *J BUON*, vol. 23, no. 3, pp. 763–768, 2018.

[30] J. Zhang *et al.*, "Tannic acid mediated induction of apoptosis in human glioma Hs 683 cells," *Oncol Lett*, Mar. 2018, doi: 10.3892/ol.2018.8197.

[31] N. SP *et al.*, "Tannic Acid Inhibits Non-small Cell Lung Cancer (NSCLC) Stemness by Inducing G_0/G_1 Cell Cycle Arrest and Intrinsic Apoptosis," *Anticancer Res*, vol. 40, no. 6, pp. 3209–3220, Jun. 2020, doi: 10.21873/anticanres.14302.

[32] F. Nie *et al.*, "Apoptotic effect of tannic acid on fatty acid synthase over-expressed human breast cancer cells," *Tumor Biology*, vol. 37, no. 2, pp. 2137–2143, Feb. 2016, doi: 10.1007/s13277-015-4020-z.

[33] H. Li, S. Krstin, and M. Wink, "Modulation of multidrug resistant in cancer cells by EGCG, tannic acid and curcumin," *Phytomedicine*, vol. 50, pp. 213–222, Nov. 2018, doi: 10.1016/j.phymed.2018.09.169.

[34] P. Yang, G.-B. Ding, W. Liu, R. Fu, A. Sajid, and Z. Li, "Tannic acid directly targets pyruvate kinase isoenzyme M2 to attenuate colon cancer cell proliferation," *Food Funct*, vol. 9, no. 11, pp. 5547–5559, 2018, doi: 10.1039/C8FO01161C.

[35] L. T. Ta, T. T. K. Nguyen, and H. Yoo, "Tannic acid-induced apoptosis in FaDu hypopharyngeal squamous cell carcinoma," *International Journal of Oral Biology*, vol. 44, no. 2, pp. 43–49, Jun. 2019, doi: 10.11620/IJOB.2019.44.2.43.

[36] J. Sánchez-Carranza *et al.*, "Phenolic Compounds Isolated from Caesalpinia coriaria Induce S and G2/M Phase Cell Cycle Arrest Differentially and Trigger Cell Death by Interfering with Microtubule Dynamics in Cancer Cell Lines," *Molecules*, vol. 22, no. 4, p. 666, Apr. 2017, doi: 10.3390/molecules22040666.
