# Supplementary material for: Structural characterization and evaluation of antimicrobial and cytotoxic activity of six plant phenolic acids
Source: PLoS One. 2024 Jun 17;19(6):e0299372. doi: 10.1371/journal.pone.0299372 (PMC11182523; doi:10.1371/journal.pone.0299372)
Supplement: S3 Table — (DOCX) [file pone.0299372.s005.docx]

|  | ***p*-CA** | **CA** | **RA** | **5-CQA** | **GA** | **TA** |
| --- | --- | --- | --- | --- | --- | --- |
| **Energy [Hartree]** | -573.62 | -648.72 | -1297.08 | -1297.96 | -646.53 | -6391.17 |
| **Dipole Moment [D]** | 3.3398 | 4.4838 | 5.8359 | 5.7089 | 0.4704 | 14.2333 |
