# Supplementary material for: Structural characterization and evaluation of antimicrobial and cytotoxic activity of six plant phenolic acids
Source: PLoS One. 2024 Jun 17;19(6):e0299372. doi: 10.1371/journal.pone.0299372 (PMC11182523; doi:10.1371/journal.pone.0299372)
Supplement: Attachment — Submitted filename: Kalinowska_manuscript_correct.docx [file pone.0299372.s006.docx]

**Structural characterization and evaluation of antimicrobial and cytotoxic activity of six plant phenolic acids**

M. Kalinowska*^1^, R. Świsłocka^1^, E. Wołejko^1^, A. Jabłońska-Trypuć^1^, U. Wydro^1^, M. Kozłowski^1^, K. Koronkiewicz^1^, J. Piekut^2^, W. Lewandowski^1^

^1^ Department of Chemistry, Biology and Biotechnology, Institute of Environmental Engineering and Energetics, Faculty of Civil Engineering and Environmental Sciences, Bialystok University of Technology, Wiejska 45E Street, 15-351 Bialystok, Poland

^2^ Department of Agri-Food Engineering and Environmental Management, Institute of Environmental Engineering and Energetics, Faculty of Civil Engineering and Environmental Sciences, Bialystok University of Technology, Wiejska 45E Street, 15-351 Bialystok, Poland

* Corresponding author

E-mail: m.kalinowska@pb.edu.pl (M.K.)

**Abstract**

Phenolic acids still gain significant attention due to their potential antimicrobial and cytotoxic properties. In this study, we have investigated the antimicrobial of six phenolic acids, namely chlorogenic, caffeic, *p*-coumaric, rosmarinic, gallic and tannic acids in the concentration range 0.5-500 µM, against *Escherichia coli* and *Lactobacillus rhamnosus*. The antimicrobial activity was evaluated using the 3-(4,5-dimethylthiazol-2-yl)-2,5-diphenyltetrazolium bromide colorimetric assay. Additionally, the cytotoxic effects of these phenolic acids on two cancer cell lines, the colorectal adenocarcinoma Caco-2 cell line and Dukes' type C colorectal adenocarcinoma DLD-1 cell line was examined. To further understand the molecular properties of these phenolic acids, quantum chemical calculations were performed using the Gaussian 09W program. Parameters such as ionization potential, electron affinity, electronegativity, chemical hardness, chemical softness, dipole moment, and electrophilicity index were obtained. The lipophilicity properties represented by logP parameter was also discussed. This study provides a comprehensive evaluation of the antimicrobial and cytotoxic activity of six phenolic acids, compounds deliberately selected due to their chemical structure. They are derivatives of benzoic or cinnamic acids with the increasing number of hydroxyl groups in the aromatic ring. The integration of experimental and computational methodologies provides a knowledge of the molecular characteristics of bioactive compounds and partial explanation of the relationship between the molecular structure and biological properties. This knowledge aids in guiding the development of bioactive components for use in dietary supplements, functional foods and pharmaceutical drugs.

**Introduction**

There has been a growing interest in natural compounds as potential pharmaceutics or chemical agents including antioxidant or preservatives that can be applied in food industry. Evidence gathered by researchers suggests that a diet abundant in foods derived from plants, can substantially decrease the susceptibility to various types of cancers, may reduce the risk of cardiovascular diseases and type 2 diabetes (1–3). One of the known natural bioactive substances are phenolic compounds, which include flavonoids, phenolic acids, or tannins (1,4). Phenolic compounds derived from carbohydrates, amino acids, and lipids, are secondary metabolites that are involved primarily in protecting plant against UV radiation, viruses, bacteria and insects. They also contribute to the aroma, colour and flavour of plant products (5). Phenolic compounds are widely recognized as potent natural antioxidants, playing a pivotal role in a diverse array of biological properties, including anti-inflammatory, anticancer, antimicrobial, antiallergic, antiviral, antithrombotic and hepatoprotective effects (6–8). In the past decade, scientific publications taking a closer look at phenolic compounds have increasingly highlighted their antibacterial and cytotoxic properties and the potential for their use. Hence, there is a great deal of co-occurrence of these keywords in many global studies (Fig 1). Phenolic acids are among the most widely distributed plant non-flavonoid phenolic compounds present in the free, conjugated-soluble and insoluble-bound forms, they are found in all food groups, they are abundant in cereals, legumes, oilseeds, fruits, vegetables, beverages and herbs.

One of the most abundant hydroxycinnamic acid derivative is chlorogenic acid (5-CQA) (S1 Fig) (9). 5-CQA is an ester of caffeic acid and (−)-quinic acid, and one of the most abundant phenolic acids, is widely distributed in fruits and vegetables like: apples, pears, carrots, or potatoes. Additionally it can be found most consumed brewages in the world wine, tea and coffee (10–12). Notably chlorogenic acid demonstrates antimicrobial activity against bacteria (Table 1) like Gram-negative *Escherichia coli* (MIC 7.06 mg/mL) (13) and Gram-positive *Alicyclobacillus acidoterrestris* (MIC 2 μg/mL) (14) and fungi *Candida albicans* with MIC (80 µg/mL) (15). 5-CQA exhibits antimicrobial activity by disrupting pore formation, the cell membrane and loss of membrane potential. It binds to the outer membrane of bacteria, leading to permeability changes and release of cytoplasmic macromolecules, resulting in cell death (16). 5-CQA possesses ability to modulate gene expression related to the immune pathway and survival of tumor cells. Its main cytotoxic effects on cancer cells are triggering apoptosis, suppressing cell proliferation, or inhibiting the cell cycle. Additionally, 5-CQA fosters formation of DNA complexes with topoisomerases, which contributes to process of apoptosis. Studies show (Table 2) lack of cytotoxicity of 5-CQA towards HaCaT cell line in concentration range of 0.15–1000 nM (10), HL-60 and Jurkat cells in concentrations of 1–100 μM (17).

**Fig 1. Co-occurrence of selected keywords in articles in 2014–2023 (search terms: “phenolic compounds”, “cytotoxicity”, “antimicrobial activity”), created with VOSviewer** (18)

Caffeic acid (CA) (S1 Fig), classified as a secondary metabolite in lignin biosynthesis, is a well-studied phenolic acid present in numerous plant species. It can be found in herbs like basil, rosemary, sage, oregano and even in vegetable like carrots, potatoes, and propolis (19). Furthermore, CA is biological active compound possessing many properties including antimicrobial, anticancerogenic, antiplatelet, antioxidative, and anti-inflammatory (20). Its antimicrobial abilities studied by Arciszewska et al (Table 1) showed inhibitory effect against *E. coli* (MIC 13 mg/mL, *B. subtilis* (MIC 18 mg/mL) and antifungal inhibiting growth of *Candida albicans* (MIC 29 mg/mL) (21). Another research showed that CA completely inhibits growth of *S. aureus, P. aeruginosa, E. coli, C. albicans,A. brasiliensis* at concentration of 5 mM (22). CA antimicrobial effect involves causing impairment in membrane function of bacterial cells. It possesses nucleophilic properties and can donate an electron pair to plasma membrane proteins and lipids, potentially leading to membrane dysfunction. CA may physically damage the cytoplasmic membrane, resulting in the leakage of intracellular components and affecting various cellular processes such as enzyme activity, proton motive force, acidification of cytoplasm, and DNA/RNA synthesis (23), (24). In terms of its anticancer activity against human cells, CA has been studied using different cell lines and assays by many researchers (Table 2). For example research conducted by Wang et all, in human leukemia cell lines MV4-11 and MOLM-14, CA does not demonstrated cytotoxic effects, however it had shown IC_50_ of 6,4 µmol/l for MOLM-13 cell line (25). CA has been investigated in various cancer cell lines, including MDAMB-231, SK-BR-3, HeLa, HT-29, Neuro 2A, PC-3, and Vero cells. In these cell lines, it exhibited IC_50_ values ranging from 104 µg/ml to 261 µg/mL, indicating its potential cytotoxic effects against various type of human cancer cells but also animal ones (26). CA shows different cytotoxic mechanism and effects depending on the cell type. HL-60 cells and human peripheral blood mononuclear cells it does not cause DNA damage, but at high concentrations, it may lead to DNA damage in human lymphocytes (17). CA's anticancer effects are attributed to its both antioxidant and pro-oxidant capacities, its chemical structure that possesses phenolic hydroxyl groups and a double bond in the carbon chain. These factors enable the elimination of free radicals and prevention of reactive oxygen species production as well its ability to stimulate the expression of P53 and P21 genes while inhibiting CDK2 gene expression, leads to cell cycle arrest in the G0/G1 phase and the inhibition of MMP-2 and MMP-9 expression, which are involved in cancer invasion and metastasis (27), (28).

*p*-Coumaric acid (*p*-CA) (S1 Fig), a natural compound, is commonly found in cherries, walnuts, onions, pear, grapes and wine either as an esterified form or as a free acid. It serves as a crucial precursor for the synthesis of various other phenolic acids, including caffeic, chlorogenic, rosmarinic, and ferulic acids (29). Experimental and clinical studies have demonstrated that *p*-CA possesses a range of beneficial properties, including antioxidant, and anti-inflammatory activities, making it highly valuable for various industries such as food, health, cosmetics and pharmaceuticals (29) (30) (31).
The antimicrobial properties of *p*-CA antimicrobial properties have attracted the attention of many researchers (Table 1). For example, Bag et al. have examined *p*-CA growth inhibitory capabilities on *Bacillus cereus and S. typhimurium* with results of respectively MIC = 0.041 mg/mL and MIC 0.104 mg/mL (32). In studies conducted by Abdel-Wareth et al. *p*-CA show no antimicrobial action against *Pseudomonas aeruginosa, Escherichia coli, Staphylococcus aureus, Aspergillus niger, or Candida albicans* but exhibited ability to inhibit growth of *Bacillus cereus* (IZ=9 mm) (33). However, research conducted by Cui et al showed that *p*-CA exhibits MIC equal to 0,6 g/L against *Staphylococcus aureus* (34). *p*-CA antibacterial mechanism was investigated by Lou et al. The results showed that p-CA caused an efflux of potassium ions from cells, indicating an increase in membrane permeability. It was revealed that *p*-CA caused membrane damage, leading to the release of cellular contents. Furthermore, their studies showed that *p*-CA can bind to DNA, potentially affecting its structure and function leading to death of a cell (35). *p*-CA exhibits significant cytotoxic abilities against various cancer cell lines (Table 2). In the MTT assay conducted by Kianmehr et al, *p*-CA demonstrated cytotoxicity against A375 cells (IC_50_ value of 800 µg/mL) and SK-MEL-37 cells exhibited an IC_50_ value of 1000 µg/mL (36). MDAMB-231 cells showed an IC_50_ value of 154 µg/mL, while SK-BR-3 cells had an IC_50_ value of 122 µg/mL. Furthermore, p-CA demonstrated its great efficacy in inhibiting the growth of HeLa cells, with an IC_50_ value of 1 µg/mL. HT-29 cells exhibited an IC_50_ value of 25 µg/mL suggesting a substantial cytotoxic effect. In same MTT assay, Vero cells derived from the kidney cells of African green monkey exhibited an IC_50_ value of 63 µg/ml (26). Karakurt et al. evaluated the cytotoxic effects of p-CA using the Alamar Blue assay. The study determined that P-CA exhibited cytotoxic abilities against DLD-1 cells with an IC_50_ value of 254.3 µg/mL Caco-2 cells with IC_50_ of 714.6 µg/mL (37).

Rosmarinic acid (RA) (S1 Fig), predominantly found in herbs such as rosemary, sage, basil, oregano, marjoram, and lemon balm (38). Some of its notable properties include neuroprotective effects, antioxidant activity, anti-inflammatory properties, antimicrobial effects, anti-angiogenic properties, heptatoprotective and anticancer potential (39–41). RA has been studied for its antimicrobial abilities (Table 1). RA showed notable antimicrobial activity against Methicillin-resistant *Staphylococcus aureus*. At a concentration of 2 mg/mL, it resulted in an inhibitory zone diameter of 11.6 mm and MIC of 10 mg/mL. Moreover, in the case of nonresistant *Staphylococcus aureus*, RA displayed an IZ diameter of 12.4 mm at a concentration of 0.2 mg/mL and MIC value 0.8 mg/mL (42). In studies conducted by Bittner Fialová et al RA also exhibited antimicrobial effects against several microorganisms. It showed a MIC value of 3.47 mg/mL both against Gramm negative and positive bacteria like against *S. aureus, E. faecalis, P. aeruginosa, K. pneumoniae* and *P. mirabilis*. Additionally, it displayed greater efficiency in inhibiting growth of *E. coli* with MIC of 1.73 mg/mL. Moreover, it showed inhibitory effects at concentrations 0.5 mg/mL against fungi *Trichoderma viridae* and *Mucor racemosus*, however the same concentration does not have inhibited growth of *Aspergillus flavus* and *Aspergillus niger* (43)*.* RA exhibits multiple mechanisms of action that contribute to its antimicrobial properties. RA cause depolymerization of the cell membrane, which results to modifications in the bacterial membrane upon contact. Consequently, this leads to a bacteriostatic effect and effectively eradicates bacterial cell membrane and their proteins. Furthermore, RA inhibits the activity of Na^+^/K^+^-ATPase in bacterial cells. In terms of antifungal mechanisms, RA may hinders mitochondrial activity, compromises membrane integrity (28). Moreover, RA displayed the ability to hinder cell attachment and eliminate established biofilms to some extent, this is partially attributed to its ability to decrease exopolysaccharide production (44). RA demonstrated an IC_50_ value of 2.9 µmol/l for the MOLM-13 cell line, the MOLM-14 cell line showed an IC_50_ value of 7.1 µmol/l (45). Furthermore, RA exhibited significant cytotoxicity against breast cancer cell lines. For MCF-7 cells, the IC_50_ value was 801 µg/ml, indicating its ability to inhibit cell proliferation. MDAMB-231, SK-BR-3, and HeLa cells also showed notable susceptibility to RA, with IC_50_ values of 121 µg/ml, 147 µg/ml, and 139 µg/ml, respectively. Similarly, the IC_50_ values for RA against HT-29, Neuro 2A, PC-3, and Vero cells were 150 µg/ml, 171 µg/ml, 384 µg/ml, and 388 µg/ml, respectively (26). Numerous mechanisms have been suggested to explain the anticancer effects of RA. One of them is demonstrated in anti-prostate cancer properties by inhibiting the colony formation, viability, and spheroid creation of cells. This is achieved by RA ability to inhibit HDAC2, leading to cell cycle arrest and apoptosis mediated by p53 protein (46). RA exhibited a dose-dependent inhibition of CCRF-CEM and CEM/ADR5000 cells, while displaying reduced cytotoxicity towards normal lymphocytes. Furthermore, RA-induced MMP dysfunction led to the activation of PARP cleavage and caspase-independent apoptosis. It was found that RA blocked the translocation of p65 from the cytosol to the nucleus (47). The primary pathway for RA-mediated apoptosis was found to be the PI3K/AKT pathway. RA downregulated the PI3K/AKT/mTOR signaling pathway, resulting in apoptosis induction, and inhibition of epithelial-mesenchymal transition (48).

Gallic acid (S1 Fig) (GA) is the most popular of trihydroxybenzoic acids that is found in the leaves of pomegranate bark, gall nuts, Indian gooseberry, witch hazel, sumac, tea leaves, oak bark, and many other plants, both in free form and as part of tannin molecules. It occurs in abundance in processed beverages such as red wine or green tea. Its most notable application lies within the pharmaceutical industry where GA plays a role as a primary raw material for the synthesis of essential compounds for broad-spectrum of antibiotic (49). GA has been found exhibiting anti-inflammatory potential and can reduce oxidative damage by scavenging reactive oxygen species (50). GA exhibits significant antimicrobial abilities against a wide range of microorganisms, including bacteria and fungi (Table 1). In studies conducted by Kalinowska et al it displayed a MIC of 3 mg/mL against *E. coli, B. subtilis, S. enteritidis*, and *C. albicans*, however *S. aureus* was more resistant to GA antibacterial abilities with MIC 6 mg/mL (51). Furthermore, it generates an IZ of 11 mm against *P. fluorescens*, 13.2 mm against *P30-4*, 15 mm against *P. fragi*, and 14.8 against *P. putida* (52). In the case of fungal infections, GA demonstrates its effectiveness by inhibiting the growth of S. mutans and *P. aeruginosa*, with MIC values of 62.5 μg/mL and 100 μg/mL, respectively (53). Previous studies attributes antimicrobial abilities of GA to various mechanisms. One of them is the inhibition of efflux pumps, which contribute to antimicrobial resistance in bacteria (54). GA might cause impairing in the permeability of cellular membranes, which might lead to DNA degradation, impairment of gene expression and cell signaling, disruption of energy metabolism, and ATP depletion (55). Not only GA can influence integrity of the cell membrane but also alter its charge, permeability, hydrophobicity. Furthermore, it can disintegrate the outer membrane of Gram-negative bacteria through chelation of divalent cations. GA exhibit excitatory activity on topoisomerase IV-mediated DNA cleavage and may also disrupt actions of bacterial dihydrofolate reductase (56). GA has been evaluated for its cytotoxicity in various cell lines using different assays (Table 2). In the research conducted by Pham et al. on A2780, H460, A431, MCF-7, and MCF10A cell lines using the MTT assay, the concentration of 50 µM GA did not exhibit any cytotoxic properties after 72 h of incubation (57). However, studies by Rezaei-Seresht et al. on breast cancer MCF-7 line showed that GA possess cytotoxic activity against this specific cell line with IC_50_ value of 18 µg/ml (27). GA exhibits cytotoxic abilities through various mechanisms, particularly in cancer cells. GA has been found to increase the expression of p53 and disrupts mitochondrial membranes, which may lead to cytochrome C release, caspase-3 activation, and subsequent apoptotic cell death. Additionally, GA can mediate in DNA damage-related regulations and overexpression of the P21 gene. The P21 can promote cellular differentiation and inhibition of cell proliferation.

Tannic acid (TA) (S1 Fig) is a naturally occurring plant polyphenol that can be found in various plants notably in certain tree species such as sumac, oak, and myrobalan, particularly their barks, are rich sources of it. Additionally, tannic acid can be found in galls formed on plants (58). It has a rich history of medicinal use, including the treatment of diarrhea, burns, and rectal disorders. Tannic acid is formed by the esterification of gallic acid dimers with the hydroxyl groups of D-glucose (59). It has been widely recognized for its antioxidant, anticarcinogenic, and antimutagenic activities. Additionally, it has been associated with various health benefits, including modulating immune responses, reducing allergen levels, and accelerating blood clotting (59,60) (61). Tannic acid exhibits antibacterial abilities against various bacterial strains. The minimum inhibitory concentration (MIC) of tannic acid in studies conducted by Sahiner et al. is as follows: 60 μg/ml for P. aeruginosa, 60 μg/ml for *B. subtilis*, 40 μg/ml for *S. aureus* and *C. albicans* 70 μg/ml. However, in the same study they have TA expressed no antimicrobial activity against *E. coli* (61). Tannic acid demonstrates inhibitory effects against *E. coli* with an average diameter of 13.33 mm and against S. aureus with a diameter of 10.16 mm (62). Moreover, tannic acid exhibits a moderately inhibitory effect against *S. Typhi* 400 μg/ml MQIC. In the case of methicillin-resistant Staphylococcus aureus, tannic acid shows an MIC of 64 μg/ml (63). TA possesses ability to penetrate the bacterial cell wall and reach the internal membrane, which disturbs bacterial metabolism, and might cause cell destruction by DNA leakage. Moreover, tannic acid limits bacteria growth by impeding the absorption of sugars and amino acids by bacteria (64). TA demonstrates cytotoxic abilities against various cancer cell lines (Table 2). In the MTT assay conducted by Zhang et al., tannic acid exhibits an IC_50_ of 4.2 µM in Hs 683 cells (65). In research of Nie et al. Tannic acid demonstrates an IC_50_ of 2.5 µM for MDA-MB-231 cells and an IC_50_ value of 4 µM for MCF-7 cells, indicating its cytotoxicity against this breast cancer cell line (66). Tannic acid shows an IC_50_ of 46.29 µM in Caco-2 cells, showing it possesses cytotoxic abilities against this colorectal cancer cell line (67). In case of DLD-1 cells, the IC_50_ value of TA is 53.6 µM (68). Furthermore, tannic acid demonstrates cytotoxic abilities against several other cancer cell lines. It exhibits an IC50 of 43.1 µM in HCT-116 cells (68), 50 µM in FaDu cells (69). TA exhibits various cytotoxic mechanisms against cancers cells. In cells of breast cancer, TA causes caspase-mediated apoptosis. It suppresses intracellular fatty acid synthase activity and decreases fatty acid synthase expression without affecting normal breast epithelial cells (66). TA inhibits the epidermal growth factor receptor pathway and enhances the p38/STAT1 pathway which induces G1 arrest and activation of the apoptotic pathway (70). TA reduces viability, metastatic potential, and stemness in cancer cells, inducing cell cycle arrest and caspase-dependent mitochondrial-mediated apoptosis (71). TA exhibits antiangiogenic properties, inhibiting migration and tumor formation induced by chemokine CXCL12, which is involved in tumor microenvironment communication (72). It also possesses ability to suppresses epithelial-to-mesenchymal transition by binding to transforming growth factor-β1 (71).

The purpose of this study is twofold. Firstly, it aims to evaluate the antimicrobial activity of the six phenolic acids against of clinical importance. Secondly, the research investigates the cytotoxic effects of the phenolic acids on colorectal adenocarcinoma cell lines. Additionally, the utilization of quantum chemical calculations and lipophilicity determination provides a comprehensive understanding of the physicochemical properties. This knowledge serves as a guide for the development of bioactive components that can be utilized in various applications, including dietary supplements, functional foods, and pharmaceutical drugs. In this study, we will evaluate the antimicrobial activity of these six phenolic acids against a bacterial strains of *Escherichia coli* and *Lactobacillus rhamnosus* based on 3-(4,5-dimethylthiazol-2-yl)-2,5-diphenyltetrazolium bromide colorimetric assay. Studies of antimicrobial activity aim to determine the suitability of the tested compounds as potential preservatives with antimicrobial properties against selected food pathogens (*E.coli*). Moreover, the studies on the effect of the analysed compounds on *L.* *rhamnosus* were conducted, which are part of the human intestinal microflora to exclude any negative effects on beneficial probiotic bacteria. We will investigate those phenolic acids cytotoxic effects of on two cancer cell lines, the colorectal adenocarcinoma Caco-2 cell line and Dukes' type C colorectal adenocarcinoma DLD-1 cell line in order to analyze potential anticancer activity of studied compounds. Moreover, quantum chemical calculations were conducted in the Gaussian 09W program to provide parameters like ionization potential, electron affinity, electronegativity, chemical hardness, chemical softness, dipole moment and electrophilicity index. Additionally, we will present lipophilicity values of those compounds represented by logP calculated by ADC/labs Percepta program.

**Table 1. Antimicrobial effects of phenolic acids on microbial strains (minimal inhibitory concentration (MIC) and inhibition zone (IZ)**

| **Compound** | **Value** | **Unit** | **Standard** | **Microorganism Strain** | **References** |
| --- | --- | --- | --- | --- | --- |
| **GA** | 17,6 | mM | MIC* | *E. coli* | (51) |
|  | 35,3 |  |  | *S. aureus* |  |
|  | 17,6 |  |  | *B. subtilis* |  |
|  | 17,6 |  |  | *S. enteritidis* |  |
|  | 17,6 |  |  | *C. albicans* |  |
|  | 11.0 ± 0.35 | mm | IZ | *P. fluorescens* | (52) |
|  | 13.2 ± 0.19 |  |  | *P30-4* |  |
|  | 15.0 ± 0.9 |  |  | *P. fragi* |  |
|  | 14.8 ± 0.4 |  |  | *P.putida* |  |
|  | 29,4 | mM | MIC | *P. fluorescens* |  |
|  | 29,4 |  |  | *P30-4* |  |
|  | 14,7 |  |  | *P. fragi* |  |
|  | 14,7 |  |  | *P. putida* |  |
|  | 0,37 | mM | MIC | S. mutans | (53) |
|  | 0,59 |  |  | *P. aeruginosa* |  |
|  | 1,47 | mM | MIC | M. haemolytica | (54) |
|  | 2,93 |  |  | P. multocida |  |
| **5-CQA** | 28,22 | mM | MIC | *Staphylococcus aureus* | (73) |
|  | 28,22 |  |  | *Enterococcus faecium* |  |
|  | 28,22 |  |  | *Escherichia coli* |  |
|  | 28,22 |  |  | *Proteus vulgaris* |  |
|  | 28,22 |  |  | *Pseudomonas aeruginosa* |  |
|  | 14,11 |  |  | *Klebsiella pneumoniae* |  |
|  | 28,22 |  |  | *Candida albicans* |  |
|  | 19,93 | mM | MIC | *E. coli* | (13) |
|  | ˃19,93 |  |  | *Bacillus sp.* |  |
|  | ˃19,93 |  |  | *Staphylococcus Epidermidis* |  |
|  | ˃19,93 |  |  | *Streptococcus Pyogenes* |  |
|  | >39,82 |  |  | *Candida sp.* |  |
|  | 14.5+ 0.50 | mm | IZ | *Alicyclobacillus acidoterrestris* | (14) |
|  | 0,006 | mM | MIC |  |  |
|  | 11,29 | mM | MBC |  |  |
|  | 0,23 | mM | MIC | *Candida albicans ATCC 90028* | (15) |
|  | 100 | % | Inhibitory activity for C=5 [mM] | *Staphylococcus aureus CCM 4516* | (22) |
|  | 100 |  |  | *Pseudomonas aeruginosa CCM 1961* |  |
|  | 100 |  |  | *Escherichia coli CCM 4517* |  |
|  | 100 |  |  | *Candida albicans CCM 8215* |  |
|  | 100 |  |  | *Aspergillus brasiliensis CCM 8222* |  |
| **CA** | 1,39 | mM | MIC | *E. coli* | (28) |
|  | >2,78 |  |  | *Bacillus sp.* |  |
|  | >2,78 |  |  | *Staphylococcus epidermidis* |  |
|  | >2,78 |  |  | *Streptococcus pyogenes* |  |
|  | >5,55 |  |  | *Candida sp.* |  |
|  | 1,42 | mM | MIC | *S. aureus ATCC 25923* | (74) |
|  | 1,42 |  |  | *S. aureus ATCC 43300* |  |
|  | 1,42 |  |  | *S. aureus ATCC 6538* |  |
|  | 100 | % | Inhibitory activity for C = 5 [mM] | *Staphylococcus aureus CCM 4516* | (22) |
|  | 50-99 |  |  | *Pseudomonas aeruginosa CCM 1961* |  |
|  | 100 |  |  | *Escherichia coli CCM 4517* |  |
|  | 100 |  |  | *Candida albicans CCM 8215* |  |
|  | 100 |  |  | *Aspergillus brasiliensis CCM 8222* |  |
|  | 72,16 | mM | MIC | *Escherichia coli* | (21) |
|  | 99,91 |  |  | *Bacillus subtilis* |  |
|  | 160,97 |  |  | *Candida albicans* |  |
|  | 5,68 | mM | MIC | *S. aureus* | (75) |
| **p-CA** | 0,25 | mM | MIC | *Bacillus cereus* | (76) |
|  | 0,63 |  |  | *S. typhimurium* |  |
|  | - | mM | IZ | *Pseudomonas aeruginosa* | (33) |
|  | - |  |  | *Escherichia coli* |  |
|  | 9±0.6 |  |  | *Bacillus cereus* |  |
|  | - |  |  | *Staphylococcus aureus* |  |
|  | - |  |  | *Aspergillus Niger* |  |
|  | - |  |  | *Candida albicans* |  |
|  | 1,22 | mM | MIC | *Alicyclobacillus acidoterrestris* | (77) |
|  | >9,75 |  | MBC |  |  |
|  | 30,48 | mM | MIC | *C. sakazakii N112* | (78) |
|  | 15,24 |  |  | *C. sakazakii N13* |  |
|  | 15,24 |  |  | *C. sakazakii ATCC* |  |
|  | 3,66 | mM | MIC | *Staphylococcus aureus* | (34) |
| **RA** | 0,69 | mM | MIC | *E. coli* | (28) |
|  | >1,39 |  |  | *Bacillus sp.* |  |
|  | >1,39 |  |  | *Staphylococcus epidermidis* |  |
|  | >1,39 |  |  | *Streptococcus pyogenes* |  |
|  | 11.6±0.4 | mm | IZ C = 2 [mg/ml] | *Methicillin-resistant Staphylococcus aureus* | (42) |
|  | 27,75 | mM | MIC | *Methicillin-resistant Staphylococcus aureus* |  |
|  | 12.4±0.4 | mm | IZ C = 0.2 [mg/ml] | *S. aureus* |  |
|  | 2,22 | mM | MIC | *S. aureus* |  |
|  | 3.47 | mM | MIC | *S. aureus CCM 4750* | (43) |
|  | 3.47 |  |  | *E. faecalis CCM 4224* |  |
|  | 3.47 |  |  | *P. aeruginosa CCM 3955* |  |
|  | 3.47 |  |  | *K.pneumoniae CCM 4415* |  |
|  | 1.73 |  |  | *E. coli CCM 3954* |  |
|  | 3.47 |  |  | *P. mirabilis CCM 7188* |  |
|  | 0.5 |  |  | *Trichoderma viridae* |  |
|  | >0.5 |  |  | *Aspergillus flavus* |  |
|  | >0.5 |  |  | *Aspergillus Niger* |  |
|  | 0.5 |  |  | *Mucor racemosus* |  |
|  | 1,73 |  |  | *Candida albicans CCM 90028* |  |
| **TA** | - | mM | MIC | *E. coli* | (61) |
|  | 0,04 |  |  | *P. aeruginosa* |  |
|  | 0,04 |  |  | *B. subtilis* |  |
|  | 0,02 |  |  | *S. aureus* |  |
|  | 0,04 |  |  | *C. albicans* |  |
|  | 13.33 ± 0.57 | mm | IZ | *E.coli* | (79) |
|  | 10.16 ± 1.04 |  |  | *S. aureus* |  |
|  | 30 | mm | IZ | *E.coli* | (62) |
|  | 32 |  |  | *S. aureus* |  |
|  | 0,24 | mM | MQIC | *S.Typhi* | (80) |
|  | - | - | IZ | *S.Paratyphi* |  |
|  | - | - | IZ | *S.Typhi* |  |
|  | - | - | IZ | *S.Paratyphi* |  |
|  | 0,02-0,09 | mM | MIC | *Staphylococcus aureus* | (81) |
|  | 0,04 | mM | MIC | MRSA | (63) |

*MIC – minimal inhibitory concentration, IZ – inhibition zone, MBC – minimum bactericidal concentration, MQIC – minimal quorum inhibitory concentration, mm – milimeter, mM – milimolar concentration, % – percentage

**Table 2. Effects of phenolic acids on cell lines in in vitro assays**

| **Compound** | **Relation** | **Value** | **Unit** | **Standard** | **Assay** | **Cell Line** | **Source** |
| --- | --- | --- | --- | --- | --- | --- | --- |
| **GA** | > | 50 | µM | GI _50_ | MTT | A2780 | (57) |
|  | > | 50 |  |  |  | H460 |  |
|  | > | 50 |  |  |  | A431 |  |
|  | > | 50 |  |  |  | MCF-7 |  |
|  | > | 50 |  |  |  | MCF10A |  |
|  | = | 0,12 | mM | IC _50_ | MTT | T47D | (82) |
|  | = | 29.92 | % | The inhibition percentage | MTT within 4 h in GArGO (50 µg/ml) alone treated samples | IP15 | (83) |
|  | = | 11,45 | mM | IC _50_ | MTT | HeLa cells | (84) |
|  | = | 0,06 | mM | IC _50_ | MTT | MRC-5 | (85) |
|  | = | 0,26 | mM | IC _50_ | MTT | MDA-MB-231 | (86) |
|  | = | 0,11 | mM | IC _50_ | MTT | MCF-7 | (27) |
|  | = | 0,11 | mM | IC_50_ | AlamarBlue and CCK08 assays | A2780S | (87) |
|  | = | 0,21 |  |  |  | A2780CP |  |
|  | = | 0,29 |  |  |  | HOSE6-3 |  |
|  | = | 0,06 | mM | IC _50_ | MTT | Caco-2 | (88) |
|  | = | 304,35 | mM | IC _50_ | cell counting kit-8 (CCK-8) assay | A549 | (89) |
| **5-CQA** | = | 27,66 | mM | IC _50_ | cell counting kit-8 (CCK-8) assay | A549 | (90) |
|  | = | 758 ± 19.09 | µM | EC _50_ | LDH assay | Caco-2 | (91) |
|  | = | 6,36 | % | Kill % | PI assay for 200 μM | U2OS | (92) |
|  | = | 5,37 |  |  |  | MG-63 |  |
|  | = | 71% | % | cell number reduction | MTT for 400 μM | Saos‐2 | (93) |
|  | = | 40 |  |  |  | MG‐63 |  |
|  | = | 74 |  |  |  | U2OS |  |
|  | = | 40 | μM | IC _50_ | CCK-8 assay | A498 | (94) |
|  | - | - | - | IC _50_ |  | HEK293 |  |
|  | = | 15 | % | apoptotic rate | Annexin V/PI dual staining assay for 20 µM | MDA-MB-23 | (95) |
|  | = | 23 |  |  |  | 4T1 |  |
|  | = | 952±32.5 | μM | IC_50_ | iCELLigence System | MCF-7 | (96) |
|  | = | 940±21.2 |  |  |  | SK-BR-3 |  |
|  | = | 590.5±10.6 |  |  |  | MDA-MB-231 |  |
|  | = | 882.5±12.0 |  |  |  | MDA-MB-468 |  |
|  | = | 1095±121.6 |  |  |  | BT-20 |  |
| **CA** | > | 10 | μM | IC_50_ | Antiproliferative activity against human cells by CellTiter-Glo assay | MV4-11 | (45) |
|  | > | 10 | μM | IC_50_ |  | MOLM-14 |  |
|  | = | 6.4 | μM | IC_50_ |  | MOLM-13 |  |
|  | = | 49,4 | mM | IC_50_ | cell counting kit-8 (CCK-8) assay | A549 cells | (90) |
|  | = | 76 | μM | IC_50_ | Neuroprotection against amyloid beta (25 to 35)-induced cell death in rat PC12 cells pre-incubated for 3 h followed by amyloid beta addition measured after 24 h by MTT assay | PC-12 | (97) |
|  | = | 102.98 | µM | IC_50_ | XTT -NR-SRB assay | MCF-7 | (98) |
|  | = | 83.47 |  |  | SRB assay |  |  |
|  | = | 84.87 |  |  | NR assay |  |  |
|  | = | 1 | mM | IC_50_ | MTT | U118MG | (99) |
|  | = | 4 |  |  |  | DLD-1 |  |
|  | = | 826,59 | µM | EC _50_ | MTT | HCT116 | (100) |
|  | = | 1.355 | mM | IC _50_ | MTT | HEp-2 | (101) |
|  | = | 1,50 | mM | IC_50_ | MTT | MCF7 | (26) |
|  | = | 0,68 |  |  |  | MDAMB-231 |  |
|  | = | 1,45 |  |  |  | SK-BR-3 |  |
|  | = | 0,86 |  |  |  | HeLa |  |
|  | = | 0,88 |  |  |  | HT-29 |  |
|  | = | 0,58 |  |  |  | Neuro 2A |  |
|  | = | 1,35 |  |  |  | PC-3 |  |
|  | = | 0,60 |  |  |  | Vero |  |
|  | = | 460 ± 21.88 | µM | EC _50_ | MTT | Caco-2 | (91) |
| **p-CA** | = | 4,88 | mM | IC_50_ | MTT | A375 | (36) |
|  | = | 6,10 | mM |  |  | SK-MEL-37 |  |
|  | = | 603.4 | µM | IC_50_ | MTT | 786-O | (102) |
|  | = | 3.36 | mM | IC_50_ | MTT | AGS | (103) |
|  | = | 1,39 | mM | IC_50_ | MTT | MCF-7 | (26) |
|  | = | 0,94 |  |  |  | MDAMB-231 |  |
|  | = | 0,74 |  |  |  | SK-BR-3 |  |
|  | = | 0,01 |  |  |  | HeLa |  |
|  | = | 0,15 |  |  |  | HT-29 |  |
|  | = | 0,81 |  |  |  | Neuro 2A |  |
|  | = | 0,84 |  |  |  | PC-3 |  |
|  | = | 0,38 |  |  |  | Vero |  |
|  | = | 223 | µM | IC_50_ | Alamar Blue assay | DLD-1 | (104) |
|  | = | 1.6 | mM |  |  | Caco-2 |  |
| **RA** | = | 2.9 | µM | IC_50_ | Antiproliferative activity against human cells by CellTiter-Glo assay | MOLM-13 | (45) |
|  | > | 10 | µM | IC_50_ |  | MV4-11 |  |
|  | = | 7.1 | µM | IC_50_ |  | MOLM-14 |  |
|  | = | 2,22 | mM | IC_50_ | MTT | MCF-7 | (26) |
|  | = | 0,34 |  |  |  | MDAMB-231 |  |
|  | = | 0,41 |  |  |  | SK-BR-3 |  |
|  | = | 0,39 |  |  |  | HeLa |  |
|  | = | 0,42 |  |  |  | HT-29 |  |
|  | = | 0,47 |  |  |  | Neuro 2A |  |
|  | = | 1,07 |  |  |  | PC-3 |  |
|  | = | 1,08 |  |  |  | Vero |  |
|  | = | 34.6 | µM | IC_50_ | MTT | OCVAR-3 | (105) |
| **TA** | = | 4.2 | µM | IC_50_ | MTT | Hs 683 | (65) |
|  | = | 50 | µM | IC_50_ | Crystal violet assay | A549 | (71) |
|  | = | 2.5 | µM | IC_50_ | MTT | MDA-MB-231 | (66) |
|  | = | 4 |  |  |  | MCF-7 |  |
|  | = | 46.29 ± 1.86 | µM | IC_50_ | MTT | Caco-2 | (67) |
|  | = | 53,6 | µM | IC_50_ | MTT | DLD-1 | (68) |
|  | = | 43.1 | µM |  |  | HCT-116 |  |
|  | = | 50 | µM | IC_50_ | MTT | FaDu | (69) |
|  | = | 13 ± 2 | µM | IC_50_ | CellTiter 96^®^ AQueous One Solution Cell Proliferation Assay kit | CaSki | (106) |
|  | = | 22 ± 3 |  |  |  | HeLa |  |
|  | = | 12.9 ± 1.8 |  |  |  | PC3 |  |
|  | = | 23 ± 0.8 |  |  |  | Hep G2 |  |
|  | = | 11 ± 1.2 |  |  |  | Hep3B |  |
|  | = | 24 ± 0.2 |  |  |  | IHH |  |

**Materials and methods**

**Reagents**

Dulbecco’s modified Eagle’s medium (DMEM) with 4.5 mg/mL (25 mM) of glucose with Glutamax, penicillin, streptomycin, trypsin–EDTA, FBS (Fetal Bovine Serum) Gold, and PBS (Phosphate Buffer Saline) (without Ca and Mg) were provided by Gibco (San Diego, CA, USA). MTT reagent was purchased from Sigma-Aldrich.

**Quantum-chemical calculations**

The density functional theory (DFT) calculations were performed in the gas phase with approximation of the isolated molecules using the Gaussian 09 (107) program running on PC computer and Gauss View (108) molecular visualization program. To calculate the geometrical structure of the compounds the B3LYP method with 6-311++G(d,p) basis set was used. The exception was represented by a molecule of tannic acid, where the HF631/3-21G method was employed. The optimized structures were possessed the minimum in the energy with no imaginary infrared wavenumbers.

**Lipophilicity**

Using the ACD/Labs program, calculations were performed to determine the partition coefficients in the octan-1-ol/water solvent system and the pKa values.

**Bacterial Strains**

*Escherichia coli* (ATCC 25922) and *Lactobacillus rhamnosus* (ATCC 53103) were obtained from the American Type Culture Collection (Manassas, VA, USA). *E. coli* (Gram-negative bacteria) and *L. rhamnosus* (Gram-positive bacteria) were grown overnight in Mueller Hinton II Broth at 37 °C. The next day, the overnight cultures were diluted in fresh MH II Broth to obtain 10^8^ CFU/mL (CFU – colony forming units). For the antimicrobial activity was used the inoculum where the suspension of *E. coli* and *L. rhamnosus* cells was 10^6^ CFU/mL.

**Determining Antimicrobial Activity Assay**

Antimicrobial activity of tested *p*-coumaric acid (*p*-CA), caffeic acid (CA), gallic acid (GA), chlorogenic acid (5-CQA), rosmarinic acid (RA) and tannic acid (TA) against of *E. coli* and *L. rhamnosus* were calculated based on 3-(4,5-dimethylthiazol-2-yl)-2,5-diphenyltetrazolium bromide (MTT) colorimetric assay. The MTT assay was conducted according to Jablonska-Trypuc et al (2019) (109). The final concentrations of tested compounds in each well were: 500 µM, 250 µM, 125 µM, 62.5 µM, 31.25 µM, 15.63 µM, 7.81 µM, 3.91 µM and 0.98 µM. The selected concentrations correspond to the levels of detected tested compounds in the human body as a result of the consumption of products rich in phenolic compounds

**Cell culture**

The influence of 5-CQA, CA, *p*-CA, GA, TA and RA was studied in colorectal adenocarcinoma Caco-2 cell line and Dukes' type C colorectal adenocarcinoma DLD-1 cell line, which were obtained from American Type Culture Collection (ATCC). Caco-2 cells and DLD-1 cells were cultured in DMEM (Gibco) supplemented with 10% FBS (Gibco), penicillin (100 U/mL), and streptomycin (100 μg/mL) at 37°C in a humified atmosphere of 5% CO_2_ in air.

**Cytotoxicity assay**

The cytotoxicity assay was performed according to the method of Carmichael using MTT reagent (Carmichael, et al., 1987). Caco-2 and DLD-1 cells were seeded in 96-well plate at a density of 1x10^4^ cells/well. 5-CQA, CA, *p*-CA, RA, GA and TA were stored in a refrigerator at temperature 4°C and the stock solution was prepared by dissolving it in TrisHCl buffer prior analysis. The compounds were added to the cultured cells and the final concentration was in the range of 0.5µM, 1µM, 5µM, 10µM, 20µM, 50µM, 100µM, 200µM, 300µM, 500µM for every studied compound. The control cells were incubated without tested compounds. After 24h and 48h the cells were washed 3 times with PBS and subsequently incubated with 10µl of MTT solution (5mg/ml in PBS) for 2 hours at 37°C in 5% CO_2_ in an incubator. Subsequently, 100µl of DMSO was added and cells were incubated in the dark for the next 2 hours. The absorbance was measured at 570nm in a microplate plate reader GloMax®-Multi Microplate Multimode Reader. The viability of DLD-1 cells and Caco-2 cells was calculated as a percentage of control cells incubated without tested compound (110).

**Statistical analysis**

All data are given as me an values ±SD (standard deviation). Differences between treatments and untreated control human cells were analyzed by one-way ANOVA, followed by Dunnett’s procedure for multiple comparisons. Significant effects are represented by p ≤ 0.05 (*), p ≤ 0.01 (**), p ≤ 0.001 (***).

**Results and discussion**

**The theoretical electronic parameters and lipophilicity**

The calculated dipole moment, the energy of the HOMO and LUMO orbitals and the electronic parameters (such as ionization potential (I), electron affinity (A), electronegativity (χ), chemical hardness (η), softness (S), chemical potential (μ) and electrophilicity (ω) indexes calculated on the basis of the HOMO and LUMO orbital’s energy) (111) were obtained for the studied compounds (Fig. 2). The parameters are used to predicting the electronic charge transfer within the molecule, reactivity, and stability of the compounds (Tables 3 and 4) (111). Because for tannic acid the electronic parameters were obtained at different theoretical level than for the other ligands, they can’t be directly compared within the whole series of the compounds.

**Table 3. Values of dipole moment and energy calculated for *p*-coumaric acid (*p*-CA), caffeic acid (CA), gallic acid (GA), chlorogenic acid (5-CQA), rosmarinic acid (RA) and tannic acid (TA)**

|  | ***p*-CA** | **CA** | **RA** | **5-CQA** | **GA** | **TA** |
| --- | --- | --- | --- | --- | --- | --- |
| **Energy** | -573.62 | -648.72 | -1297.08 | -1297.96 | -646.53 | -6391.17 |
| **Dipole Moment [D]** | 3.3398 | 4.4838 | 5.8359 | 5.7089 | 0.4704 | 14.2333 |

The HOMO-LUMO gap values increase in the following order: RA < 5-CQA < CA < p-CA < GA, indicating the decrease in the bioreactivity of studied molecules in above series. As a result, it can be inferred that among the studied compounds, rosmarinic acid is the most chemically active, while gallic acid is the least active. The ionization potential decreases and the value of the energy of HOMO orbital increases in same the series: p-CA → GA → 5-CQA → CA → RA what means the rise in the electron donating properties in the above series of ligands in the redox reactions.

**Table 4. Energy of HOMO/LUMO orbitals and other reactivity descriptors**

| **Descriptors** | **p-CA** | **CA** | **RA** | **5-CQA** | **GA** | **TA** |
| --- | --- | --- | --- | --- | --- | --- |
| **E_HOMO_ (eV)** | -6.431 | -6.252 | -5.796 | -6.281 | -6.344 | -8.554 |
| **E_LUMO_(eV)** | -2.148 | -2.114 | -2.251 | -2.198 | -1.613 | -1.805 |
| **Energy gap (eV)** | 4.283 | 4.138 | 3.545 | 4.083 | 4.731 | 6.749 |
| **Ionization potential, I=-E_HOMO_** | 6.431 | 6.252 | 5.796 | 6.281 | 6.344 | 8.554 |
| **Electron affinity, A=-E_LUMO_** | 2.148 | 2.114 | 2.251 | 2.198 | 1.613 | 1.805 |
| **Electronegativity, χ=I +A/2** | 4.289 | 4.183 | 4.023 | 4.240 | 3.978 | 5.179 |
| **Chemical potential, µ= - I+A/2** | -4.289 | -4.183 | -4.023 | -4.240 | -3.978 | -5.179 |
| **Chemical hardness, η=I-A/2** | 2.141 | 2.069 | 1.773 | 2.042 | 2.366 | 3.374 |
| **Chemical softness, S=1/2η** | 0.234 | 0.242 | 0.282 | 0.245 | 0.211 | 0.148 |
| **Electrophilicity index, ω= µ^2^/2η** | 4.296 | 4.228 | 4.566 | 4.402 | 3.345 | 3.975 |

**Fig 2. HOMO and LUMO orbitals for *p*-coumaric acid (*p*-CA), caffeic acid (CA), gallic acid (GA), chlorogenic acid (5-CQA), rosmarinic acid (RA) and tannic acid (TA)**

The presented MEP surface (Fig 3) describes the overall molecular charge distribution and allows to predict the sites for electrophilic and nucleophilic attack. The red region in the MEP plot shows an electron-rich site with electrophilic reactivity that covers the oxygen atoms in carbonyl group and phenolic OH groups. Whereas the blue color indicates an electron-deficient region which is the site of the nucleophilic attack and it is mainly located in the region of the hydrogen atom of the phenolic OH groups.

**Fig 3. Calculated electrostatic potential surfaces on the molecules for *p*-coumaric acid (*p*-CA), caffeic acid (CA), gallic acid (GA), chlorogenic acid (5-CQA), rosmarinic acid (RA) and tannic acid (TA)**

**Lipophilicity**

The logarithmic coefficient of substance distribution (log P) in the water-n-octanol system is a crucial parameter used to assess the hydrophilicity/lipophilicity of chemicals. Factors like bioaccumulation and toxicity are largely correlated with logP. Regarding most of the analyzed phenolic acids, the collected experimental data and calculated log P values are consistent with each other (Table 5). Based on the experimental logP values, the compounds can be arranged in order of increasing lipophilicity as follows: GA<CA<5-CQA<p-CA<RA<TA. Gallic acid logP experimental value 0.70 indicates it low lipophilicity. LogP experimental values for RA, 5-CQA, CA and p-CA are in the range of 1-2 this means that these compounds possess moderately lipophilic properties. The experimental logP value of 4.86 for TA indicates it’s high lipophilic properties. pKa is a measure of the acidity or basicity of a compound. In the context of lipophilicity, pKa can provide information about the ionization state of a compound at different pH values. Compounds with lower pKa values are more likely to be ionized at physiological pH (around 7.4), while compounds with higher pKa values are more likely to be in their neutral, non-ionized form. Therefore, based on experimental data and calculated values, it can be deduced that the molecular and monodeprotonated forms of GA, RA, 5-CQA, p-CA and CF are predominant within the range of physiological pH. Among those phenolic compounds 5-CQA and RA possessed lowest pKa experimental value (2.72 and 3.57 respectively), this is due to high number of the hydroxyl groups they possess. The presence of functional groups that can act as hydrogen bond donors or acceptors enhances the hydrophilicity of a compound. Compounds containing hydroxyl and carboxylic groups have the ability to form hydrogen bonds with water molecules in an aqueous environment, influencing their solubility in water. However tannic acid possesses multiple hydroxyl groups and exhibits a pKa value of 8.5. As a result, tannic acid is significantly less acidic and highly lipophilic compared to the other studied phenolic compounds. This heightened hydrophobicity and low acidity can primarily be attributed to its strong propensity to form hydrogen bonds with itself.

**Table 5. Theoretical (calculated in ACD/Labs or Percepta: logP classic, logP galas, pKa classic) and experimental logP and pKa values**

| **Compound** | **ACD/LogP Classic** | **ACD/**  **LogP Galas** | **Conssensus P** | **LogP experimental** | **Source** | **ACD/pK_a_ Classic** | **pKa experimental** | **Source** |
| --- | --- | --- | --- | --- | --- | --- | --- | --- |
| **Gallic acid** | 0.91 ± 0.33 | 0.61 | 0.69 | 0.70 | (113) | 4.3± 0.1 | 4.21 | (114) |
| **5-CQA** | -0.36 ± 0.43 | -0.30 | -0.33 | 0.72 | (115) | 3.9± 0.5 | 2.72 | (115) |
| **Caffeic acid** | 1.42 ± 0.36 | 1.32 | 1.35 | 1.15 | (116) | 4.6± 0.1 | 4.49 | (117) |
| ***p*-coumaric acid** | 1.88 ± 0.34 | 1.63 | 1.69 | 1.79 | (118) | 4.5±0.4 | 4.64 | (119) |
| **Rosmarinic acid** | 1.70 ± 0.41 | 1.54 | 1.60 | 1.60 | (115) | 2.8± 0.1 | 3.57 | (120) |
| **Tannic acid** | 13.33 ± 0.91 | 3.93 | 9.53 | 4.86 | (121) | 6.0 ± 0.5 | 8.5 | (122) |

**Antimicrobial activity**

Fig 4 shows the influence of p-CA, CA, GA, 5-CQA, RA and TA on the concentration range 0.98-500 µM on tested *E. coli* and *L. rhamnosus* strains. The obtained results are in agreement with Table 1. In the case of *E. coli,* it was observed that CA, p-CA and 5-CQA caused an insignificant increase of the bacteria growth with rising concentration of tested acids and the highest value did not exceed 16% (Fig 4). No significant effect was observed after GA and TA application on *E. coli*. For GA the average relative viability of *E. coli* was approximately of 1 to 10% compared to untreated cells. After the application of TA at the concentrations from 62.5 to 500 µM we observed a decrease relative cell viability approximately of 1 to 10% compared to the control cells. In the studied range of concentration the selected phenolic acid showed no effect on *E. coli* [49](61).

After the application of TA on *L. rhamnosus* at the lowest concentrations from 0.98 to 15.63 µM, an increase in relative cell viability of 40 to 60% was observed, while at TA concentrations from 31.25 to 500 µM there was a decrease in the relative cell viability of 20 to 80% compared to the control untreated cells. After the application of GA on *L. rhamnosus* an increase in relative cell viability with a rise in the concentration of the analyzed compound was shown. At the highest values used from 250 to 500 µM, relative viability of *L. rhamnosus* cells was at the level of 80%, compared to untreated cells.

Comparing the effect of CA, p-CA, RA and 5-CQA acids on *L. rhamnosus* strains, it was found that the analyzed acids significantly stimulated the tested bacteria strain. The application of CA and 5-CQA acids caused a significant increase of *L. rhamnosus* with increasing concentration of the tested acids and the highest value for the bacteria did not exceed 180% (Fig 4). In turn, the application of p-CA and RA to the tested bacteria strain at the highest concentration 500 µM resulted in the largest significant inhibition of growth of *L. rhamnosus*. Moreover, after the application of RA acid at the lowest concentration of 0.98 µM, the study showed the highest average relative viability, with an increase of approximately 65% as compared to the control cells. Increasing concentration of this acid caused inhibition of growth of the tested bacteria strain (109). The obtained results suggest that the concentrations of compounds did not significantly influence the viability of *E. coli*, which indicates that these compounds do not stimulate the growth of a potentially pathogenic bacterium naturally present in the human microbiome.

**Fig 4. Relative cell viability after GA, 5-CQA, CA, p-CA, RA and TA acids treatment after 24 h depending on the concentration of the tested compounds (*- p<0.05, **- p<0.01, ***- p<0.001 represent significant effects between treated and untreated bacteria followed by a Dunnett’s test and error bars represent the standard deviation ±SD).**

**Cytotoxic activity**

As it was shown in Figs 5 and 6, studied compounds did not exert significant cytotoxic effect on Caco-2 cells. 5-CQA caused increases in relative Caco-2 cell viability even in 200µM concentration. After 48h treatment decreases in studied parameter were observed in lower concentrations (from 0.5µM to 10µM). In case of p-CA no significant decreases in cell viability as compared to control untreated cells were noticed. Relatively more significant effect was observed in case of p-CA after 48h treatment, when decreases below control level were observed in all analyzed concentrations.

It should be underline that studied compounds were more effective against DLD-1 cells that towards Caco-2 cells (Figs 5 and 6). Statistically significant decreases in relative cell viability were observed under the influence of p-CA already in 0.5µM concentration. After 48h incubation 26% decline in cells viability as compared to control untreated cells was noticed. DLD-1 cells treated with RA showed decrease in cell viability already in 5µM concentration.

As shown in Figs 5 and 6 in Caco-2 cell line cytotoxic effect was observed rather after 48h incubation with both studied compounds. In DLD-1 cell line incubation with especially TA in all analyzed concentrations leads to statistically significant decreases in cell viability. However, 5µM of GA in Caco-2 cell line caused an increase in relative cell viability. Similar effect was observed in case of TA treatment after 48h incubation time in the lowest tested concentration. TA in 200 µM concentration caused almost 50% decline in Caco-2 cell viability after 48h incubation. More significant inhibitory effect of studied compounds was observed in DLD-1 cell line than in Caco-2 cell line. Decrease by about 50% was noticed in DLD-1 cells in the concentration of 200µM for both compounds. It should be also mentioned that in case of DLD-1 cell line we did not observe any increases as compared to control untreated cells. From the group of studied compounds TA, GA and RA were especially active inDLD-1 cell line. Obtained results are in accordance with literature data indicating anticancer properties of phenolic compounds [17,18]. Differences between the individual tested compounds may result from their chemical structure and thus their ability to penetrate cell membranes. The research results presented in the literature, similarly to our results, indicate differences between the tested cell lines, which are partly due to differences in the genetic and metabolic profile of cancer cells [37]. The main difference between the Caco-2 and DLD-1 lines is their origin. Caco-2 cells represent Dukes' stage B, which means that the cancer has grown into the muscular layer of the intestine, while DLD-1 cells represent Dukes' stage C, which means that the cancer has metastasized to at least one lymph node in the intestinal area. By considering gene expression in both lines, differences between both lines were identified, which translate into their response to pro-apoptotic factors(123).

**Fig 5. Relative cell viability results for Caco-2 cells exposed to the range of 5-CQA, CA, p-CA, RA, GA and TA concentrations for 24h and 48h calculated as a percentage of control, untreated cells. Each value on the graph is the mean of three independent experiments and error bars show the standard deviation (SD). * p < 0.05, ** p < 0.01 and *** p < 0.001 represent significant effects between treatments and control followed by a Dunnett’s test.**

**Fig 6. Relative cell viability results for DLD-1 cells exposed to the range of 5-CQA, CA, p-CA and RA concentrations for 24h and 48h calculated as a percentage of control, untreated cells. Each value on the graph is the mean of three independent experiments and error bars show the standard deviation (SD). * p < 0.05, ** p < 0.01 and *** p < 0.001 represent significant effects between treatments and control followed by a Dunnett’s test.**

**Conclusions**

In the study it was observed that the tested compounds did not affect the viability of *E.coli* and *L. rhamnosus* in the tested concentration range. Among the studied phenolic acid TA showed outstanding activity toward *L. rhamnosus*. TA at the concentration 31.25 to 500 µM caused a decrease in the relative cell viability of 20 to 80% compared to the control untreated cells. The cytotoxic activity of the phenolic acids was tested in the same concentration range as the antimicrobial. Based on the obtained results, it can be concluded that a significantly better response to the applied treatments was obtained in the case of the DLD-1 line than Caco-2, and the most effective in relation to both cell lines was TA. On the other hand, CA showed the lowest biological activity, especially in the Caco-2 line. The highest cytotoxic properties of TA toward DLD-1 cell line correspond with the highest lipophilicity and stability of the molecule compared to other tested phenolic compounds. Although these two parameters are important factors determining the biological activity of chemicals, the explanation of the high activity of TA is more complicated and is related to other mechanisms involved in induction of cell apoptosis like generation of reactive oxygen species, regulation of apoptotic and anti-apoptotic proteins, suppression and promotion of oncogenes or inhibition of matrix metalloproteinases. The aspect of possible induction of apoptosis is one of the most important elements of modern therapy from the point of view of eliminating cancer cells. The vast majority of the tested compounds seem to be promising potential anticancer agents, but they require further analysis at the molecular level to understand the mechanisms of their action.

**Acknowledgments**

This research was founded by National Science Centre (NCN), Poland, under the research project number 2018/29/B/NZ9/01997.

**References**

1.	Wahle KWJ, Brown I, Rotondo D, Heys SD. Plant Phenolics in the Prevention and Treatment of Cancer. In 2010. p. 36–51.

2.	Mink PJ, Scrafford CG, Barraj LM, Harnack L, Hong CP, Nettleton JA, et al. Flavonoid intake and cardiovascular disease mortality: a prospective study in postmenopausal women. Am J Clin Nutr. 2007 Mar;85(3):895–909.

3.	Kasprzak K, Oniszczuk T, Wójtowicz A, Waksmundzka-Hajnos M, Olech M, Nowak R, et al. Phenolic Acid Content and Antioxidant Properties of Extruded Corn Snacks Enriched with Kale. J Anal Methods Chem. 2018;2018:1–7.

4.	Heleno SA, Martins A, Queiroz MJRP, Ferreira ICFR. Bioactivity of phenolic acids: Metabolites versus parent compounds: A review. Food Chem. 2015 Apr;173:501–13.

5.	Kumar N, Goel N. Phenolic acids: Natural versatile molecules with promising therapeutic applications. Biotechnology Reports. 2019 Dec;24:e00370.

6.	Middleton E, Kandaswami C, Theoharides TC. The effects of plant flavonoids on mammalian cells: implications for inflammation, heart disease, and cancer. Pharmacol Rev. 2000 Dec;52(4):673–751.

7.	Rocha LD, Monteiro MC, Teodoro AJ. Anticancer Properties of Hydroxycinnamic Acids -A Review. Cancer Clin Oncol. 2012 Oct 30;1(2).

8.	Kumar N, Pruthi V. Potential applications of ferulic acid from natural sources. Biotechnology Reports. 2014 Dec;4:86–93.

9.	Padmanabhan P, Correa-Betanzo J, Paliyath G. Berries and Related Fruits. In: Encyclopedia of Food and Health. Elsevier; 2016. p. 364–71.

10.	Kalinowska M, Gryko K, Gołębiewska E, Świderski G, Lewandowska H, Pruszyński M, et al. Fe(III) and Cu(II) Complexes of Chlorogenic Acid: Spectroscopic, Thermal, Anti-/Pro-Oxidant, and Cytotoxic Studies. Materials. 2022 Oct 1;15(19):6832.

11.	Gagliardini E, Benigni A, Perico N. Pharmacological Induction of Kidney Regeneration. In: Kidney Transplantation, Bioengineering and Regeneration. Elsevier; 2017. p. 1025–37.

12.	Veljkovic E, Xia W, Phillips B, Wong ET, Ho J, Oviedo A, et al. Other Compounds From Tobacco With Potential Impact on Neurodegenerative Diseases. In: Nicotine and Other Tobacco Compounds in Neurodegenerative and Psychiatric Diseases. Elsevier; 2018. p. 83–97.

13.	Kalinowska M, Bajko E, Matejczyk M, Kaczyński P, Łozowicka B, Lewandowski W. The Study of Anti-/Pro-Oxidant, Lipophilic, Microbial and Spectroscopic Properties of New Alkali Metal Salts of 5-O-Caffeoylquinic Acid. Int J Mol Sci. 2018 Feb 4;19(2):463.

14.	Cai R, Miao M, Yue T, Zhang Y, Cui L, Wang Z, et al. Antibacterial activity and mechanism of cinnamic acid and chlorogenic acid against *Alicyclobacillus acidoterrestris* vegetative cells in apple juice. Int J Food Sci Technol. 2019 May 11;54(5):1697–705.

15.	Yun J, Lee DG. Role of potassium channels in chlorogenic acid-induced apoptotic volume decrease and cell cycle arrest in Candida albicans. Biochimica et Biophysica Acta (BBA) - General Subjects. 2017 Mar;1861(3):585–92.

16.	Lou Z, Wang H, Zhu S, Ma C, Wang Z. Antibacterial Activity and Mechanism of Action of Chlorogenic Acid. J Food Sci. 2011 Aug;76(6):M398–403.

17.	Hernandes LC, Machado ART, Tuttis K, Ribeiro DL, Aissa AF, Dévoz PP, et al. Caffeic acid and chlorogenic acid cytotoxicity, genotoxicity and impact on global DNA methylation in human leukemic cell lines. Genet Mol Biol. 2020;43(3).

18.	van Eck N, Waltman L. Software Survey: VOSviewer, a Computer Program for Bibliometric Mapping. Scientometrics. 2010;84:523–38.

19.	Meinhart AD, Damin FM, Caldeirão L, de Jesus Filho M, da Silva LC, da Silva Constant L, et al. Chlorogenic and caffeic acids in 64 fruits consumed in Brazil. Food Chem. 2019 Jul;286:51–63.

20.	Meinhart AD, Damin FM, Caldeirão L, de Jesus Filho M, da Silva LC, da Silva Constant L, et al. Study of new sources of six chlorogenic acids and caffeic acid. Journal of Food Composition and Analysis. 2019 Sep;82:103244.

21.	Arciszewska Ż, Gama S, Kalinowska M, Świderski G, Świsłocka R, Gołębiewska E, et al. Caffeic Acid/Eu(III) Complexes: Solution Equilibrium Studies, Structure Characterization and Biological Activity. Int J Mol Sci. 2022 Jan 14;23(2):888.

22.	Kyselka J, Rabiej D, Dragoun M, Kreps F, Burčová Z, Němečková I, et al. Antioxidant and antimicrobial activity of linseed lignans and phenolic acids. European Food Research and Technology. 2017 Sep 27;243(9):1633–44.

23.	Perumal S, Mahmud R, Ismail S. Mechanism of action of isolated caffeic acid and epicatechin 3-gallate from Euphorbia hirta against Pseudomonas aeruginosa. Pharmacogn Mag [Internet]. 2017;13(50):311. Available from: https://phcog.com/article/view/13/suppl-2/s311-s315

24.	Khan F, Bamunuarachchi NI, Tabassum N, Kim YM. Caffeic Acid and Its Derivatives: Antimicrobial Drugs toward Microbial Pathogens. J Agric Food Chem. 2021 Mar 17;69(10):2979–3004.

25.	Wang Q, Hu Z, Li X, Wang A, Wu H, Liu J, et al. Salviachinensines A-F, Antiproliferative Phenolic Derivatives from the Chinese Medicinal Plant Salvia chinensis. J Nat Prod. 2018;81(11).

26.	Sevimli-Gur C, Yesil-Celiktas O. Cytotoxicity screening of supercritical fluid extracted seaweeds and phenylpropanoids. Mol Biol Rep [Internet]. 2019 Aug 19;46(4):3691–9. Available from: http://link.springer.com/10.1007/s11033-019-04812-9

27.	Rezaei-Seresht H, Cheshomi H, Falanji F, Movahedi-Motlagh F, Hashemian M, Mireskandari E. Cytotoxic activity of caffeic acid and gallic acid against MCF-7 human breast cancer cells: An in silico and in vitro study. Avicenna J Phytomed. 2019;9(6):574–86.

28.	Matejczyk M, Świsłocka R, Golonko A, Lewandowski W, Hawrylik E. Cytotoxic, genotoxic and antimicrobial activity of caffeic and rosmarinic acids and their lithium, sodium and potassium salts as potential anticancer compounds. Adv Med Sci. 2018 Mar;63(1):14–21.

29.	Taofiq O, González-Paramás A, Barreiro M, Ferreira I. Hydroxycinnamic Acids and Their Derivatives: Cosmeceutical Significance, Challenges and Future Perspectives, a Review. Molecules. 2017 Feb 13;22(2):281.

30.	Kiliç I, Yeşiloğlu Y. Spectroscopic studies on the antioxidant activity of p-coumaric acid. Spectrochim Acta A Mol Biomol Spectrosc. 2013 Nov;115:719–24.

31.	Nasr G, Banaszak Holl MM, Greige-Gerges H, Hamade A, Daakour S, atallah C. P-Coumaric Acid in Poly(Amidoamine) G4 Dendrimer: Characterization and Toxicity Evaluation. SSRN Electronic Journal. 2022;

32.	Bag A, Chattopadhyay RR. Synergistic antibacterial and antibiofilm efficacy of nisin in combination with p-coumaric acid against food-borne bacteria Bacillus cereus and Salmonella typhimurium. Lett Appl Microbiol. 2017;65(5).

33.	Abdel-Wareth M, Ghareeb M. Bioprospecting certain freshwater-derived fungi for phenolic compounds with special emphasis on antimicrobial and larvicidal activity of methyl gallate and p-coumaric Acid. Egypt J Chem. 2018 Jun 22;0(0):0–0.

34.	Cui S, Ma X, Wang X, Zhang TA, Hu J, Tsang YF, et al. Phenolic acids derived from rice straw generate peroxides which reduce the viability of Staphylococcus aureus cells in biofilm. Ind Crops Prod. 2019 Nov;140:111561.

35.	Lou Z, Wang H, Rao S, Sun J, Ma C, Li J. p-Coumaric acid kills bacteria through dual damage mechanisms. Food Control. 2012 Jun;25(2):550–4.

36.	Kianmehr Z, Khorsandi K, Mohammadi M, Hosseinzadeh R. Low-level laser irradiation potentiates anticancer activity of p-coumaric acid against human malignant melanoma cells. Melanoma Res. 2020 Apr;30(2):136–46.

37.	Karakurt S, Abuşoğlu G, Arituluk ZC. Comparison of anticarcinogenic properties of viburnum opulus and its active compound P-coumaric acid on human colorectal carcinoma. Turkish Journal of Biology. 2020;44(5).

38.	Ramos-Hryb AB, Cunha MP, Kaster MP, Rodrigues ALS. Natural Polyphenols and Terpenoids for Depression Treatment: Current Status. In 2018. p. 181–221.

39.	Wang SJ, Chen Q, Liu MY, Yu HY, Xu JQ, Wu JQ, et al. Regulation effects of rosemary ( *Rosmarinus officinalis* Linn.) on hepatic lipid metabolism in OA induced NAFLD rats. Food Funct. 2019;10(11):7356–65.

40.	Fadel O, El Kirat K, Morandat S. The natural antioxidant rosmarinic acid spontaneously penetrates membranes to inhibit lipid peroxidation in situ. Biochimica et Biophysica Acta (BBA) - Biomembranes. 2011 Dec 1;1808(12):2973–80.

41.	Budzianowski J, Romaniuk-Drapała A, Kikowska M, Budzianowska A, Thiem B, Lisiak N, et al. Rosmarinic acid 4′-O-β-glucoside – A compound with prospective medicinal and cosmetological applications - its isolation from callus and root cultures of Eryngium species and biological activity. Ind Crops Prod. 2023;193.

42.	Ekambaram S, Perumal S, Balakrishnan A, Marappan N, Gajendran S, Viswanathan V. Antibacterial synergy between Rosmarinic acid and antibiotics against Methicillin resistant Staphylococcus aureus. J Intercult Ethnopharmacol. 2016;5(4):358.

43.	Bittner Fialová S, Kello M, Čoma M, Slobodníková L, Drobná E, Holková I, et al. Derivatization of Rosmarinic Acid Enhances its in vitro Antitumor, Antimicrobial and Antiprotozoal Properties. Molecules. 2019 Mar 19;24(6):1078.

44.	Ivanov M, Kostić M, Stojković D, Soković M. Rosmarinic acid–Modes of antimicrobial and antibiofilm activities of a common plant polyphenol. South African Journal of Botany. 2022 May;146:521–7.

45.	Wang Q, Hu Z, Li X, Wang A, Wu H, Liu J, et al. Salviachinensines A-F, Antiproliferative Phenolic Derivatives from the Chinese Medicinal Plant Salvia chinensis. J Nat Prod [Internet]. 2018 Nov 26 [cited 2023 Jun 13];81(11):2531–8. Available from: https://pubs.acs.org/doi/abs/10.1021/acs.jnatprod.8b00638

46.	Jang YG, Hwang KA, Choi KC. Rosmarinic Acid, a Component of Rosemary Tea, Induced the Cell Cycle Arrest and Apoptosis through Modulation of HDAC2 Expression in Prostate Cancer Cell Lines. Nutrients. 2018 Nov 16;10(11):1784.

47.	Wu CF, Hong C, Klauck SM, Lin YL, Efferth T. Molecular mechanisms of rosmarinic acid from Salvia miltiorrhiza in acute lymphoblastic leukemia cells. J Ethnopharmacol. 2015 Dec;176:55–68.

48.	Wang L, Yang H, Wang C, Shi X, Li K. Rosmarinic acid inhibits proliferation and invasion of hepatocellular carcinoma cells SMMC 7721 via PI3K/AKT/mTOR signal pathway. Biomedicine & Pharmacotherapy. 2019 Dec;120:109443.

49.	Goldberg I, Rokem JS. Organic and Fatty Acid Production, Microbial. In: Encyclopedia of Microbiology. Elsevier; 2009. p. 421–42.

50.	Erukainure OL, Sanni O, Islam MdS. Clerodendrum volubile: Phenolics and Applications to Health. In: Polyphenols: Mechanisms of Action in Human Health and Disease. Elsevier; 2018. p. 53–68.

51.	Kalinowska M, Gołębiewska E, Świderski G, Męczyńska-Wielgosz S, Lewandowska H, Pietryczuk A, et al. Plant-Derived and Dietary Hydroxybenzoic Acids—A Comprehensive Study of Structural, Anti-/Pro-Oxidant, Lipophilic, Antimicrobial, and Cytotoxic Activity in MDA-MB-231 and MCF-7 Cell Lines. Nutrients. 2021 Sep 4;13(9):3107.

52.	Sorrentino E, Succi M, Tipaldi L, Pannella G, Maiuro L, Sturchio M, et al. Antimicrobial activity of gallic acid against food-related Pseudomonas strains and its use as biocontrol tool to improve the shelf life of fresh black truffles. Int J Food Microbiol. 2018 Feb;266:183–9.

53.	Osamudiamen PM, Oluremi BB, Oderinlo OO, Aiyelaagbe OO. Trans-resveratrol, piceatannol and gallic acid: Potent polyphenols isolated from Mezoneuron benthamianum effective as anticaries, antioxidant and cytotoxic agents. Sci Afr. 2020 Mar 1;7:e00244.

54.	Rajamanickam K, Yang J, Sakharkar MK. Gallic Acid Potentiates the Antimicrobial Activity of Tulathromycin Against Two Key Bovine Respiratory Disease (BRD) Causing-Pathogens. Front Pharmacol. 2019 Jan 4;9.

55.	Sarjit A, Wang Y, Dykes GA. Antimicrobial activity of gallic acid against thermophilic Campylobacter is strain specific and associated with a loss of calcium ions. Food Microbiol. 2015 Apr;46:227–33.

56.	Nohynek LJ, Alakomi HL, Kähkönen MP, Heinonen M, Helander IM, Oksman-Caldentey KM, et al. Berry Phenolics: Antimicrobial Properties and Mechanisms of Action Against Severe Human Pathogens. Nutr Cancer. 2006 Jan;54(1):18–32.

57.	Pham HNT, Sakoff JA, Vuong Q Van, Bowyer MC, Scarlett CJ. Comparative cytotoxic activity between kaempferol and gallic acid against various cancer cell lines. Data Brief. 2018 Dec 1;21:1033–6.

58.	Khan S, Sahar A, Tariq T, Sameen A, Tariq F. Essential oils in plants: Plant physiology, the chemical composition of the oil, and natural variation of the oils (chemotaxonomy and environmental effects, etc.). In: Essential Oils. Elsevier; 2023. p. 1–36.

59.	Aelenei N, Popa MI, Novac O, Lisa G, Balaita L. Tannic acid incorporation in chitosan-based microparticles and in vitro controlled release. J Mater Sci Mater Med. 2009 May 22;20(5):1095–102.

60.	Choi J, Marshall B, Ko H, Shi H, Singh AK, Thippareddi H, et al. Antimicrobial and immunomodulatory effects of tannic acid supplementation in broilers infected with Salmonella Typhimurium. Poult Sci. 2022 Nov;101(11):102111.

61.	Sahiner N, Sagbas S, Sahiner M, Silan C, Aktas N, Turk M. Biocompatible and biodegradable poly(Tannic Acid) hydrogel with antimicrobial and antioxidant properties. Int J Biol Macromol. 2016 Jan;82:150–9.

62.	Sethulekshmi AS, Saritha A, Joseph K, Aprem AS, Sisupal SB, Nair VS, et al. Multifunctional role of tannic acid in improving the mechanical, thermal and antimicrobial properties of natural rubber-molybdenum disulfide nanocomposites. Int J Biol Macromol. 2023 Jan;225:351–60.

63.	Dong G, Liu H, Yu X, Zhang X, Lu H, Zhou T, et al. Antimicrobial and anti-biofilm activity of tannic acid against *Staphylococcus aureus*. Nat Prod Res. 2018 Sep 17;32(18):2225–8.

64.	Pandey A, Negi PS. Phytochemical composition, *in vitro* antioxidant activity and antibacterial mechanisms of *Neolamarckia cadamba* fruits extracts. Nat Prod Res. 2018 May 19;32(10):1189–92.

65.	Zhang J, Chen D, Han D, Cheng Y, Dai C, Wu X, et al. Tannic acid mediated induction of apoptosis in human glioma Hs 683 cells. Oncol Lett. 2018 Mar 7;

66.	Nie F, Liang Y, Jiang B, Li X, Xun H, He W, et al. Apoptotic effect of tannic acid on fatty acid synthase over-expressed human breast cancer cells. Tumor Biology. 2016 Feb 8;37(2):2137–43.

67.	Li H, Krstin S, Wink M. Modulation of multidrug resistant in cancer cells by EGCG, tannic acid and curcumin. Phytomedicine. 2018 Nov;50:213–22.

68.	Yang P, Ding GB, Liu W, Fu R, Sajid A, Li Z. Tannic acid directly targets pyruvate kinase isoenzyme M2 to attenuate colon cancer cell proliferation. Food Funct. 2018;9(11):5547–59.

69.	Ta LT, Nguyen TTK, Yoo H. Tannic acid-induced apoptosis in FaDu hypopharyngeal squamous cell carcinoma. International Journal of Oral Biology. 2019 Jun 30;44(2):43–9.

70.	Darvin P, Joung YH, Kang DY, Sp N, Byun HJ, Hwang TS, et al. Tannic acid inhibits <scp>EGFR</scp> / <scp>STAT</scp> 1/3 and enhances p38/ <scp>STAT</scp> 1 signalling axis in breast cancer cells. J Cell Mol Med. 2017 Apr 15;21(4):720–34.

71.	SP N, KANG DY, KIM DH, YOO JS, JO ES, RUGAMBA A, et al. Tannic Acid Inhibits Non-small Cell Lung Cancer (NSCLC) Stemness by Inducing G _0_ /G _1_ Cell Cycle Arrest and Intrinsic Apoptosis. Anticancer Res. 2020 Jun 2;40(6):3209–20.

72.	Guo F, Wang Y, Liu J, Mok SC, Xue F, Zhang W. CXCL12/CXCR4: a symbiotic bridge linking cancer cells and their stromal neighbors in oncogenic communication networks. Oncogene. 2016 Feb 18;35(7):816–26.

73.	Bajko E, Kalinowska M, Borowski P, Siergiejczyk L, Lewandowski W. 5-O-Caffeoylquinic acid: A spectroscopic study and biological screening for antimicrobial activity. LWT - Food Science and Technology. 2016 Jan;65:471–9.

74.	Kępa M, Miklasińska-Majdanik M, Wojtyczka RD, Idzik D, Korzeniowski K, Smoleń-Dzirba J, et al. Antimicrobial Potential of Caffeic Acid against *Staphylococcus aureus* Clinical Strains. Biomed Res Int. 2018 Jul 15;2018:1–9.

75.	dos Santos JFS, Tintino SR, de Freitas TS, Campina FF, de A. Menezes IR, Siqueira-Júnior JP, et al. In vitro e in silico evaluation of the inhibition of Staphylococcus aureus efflux pumps by caffeic and gallic acid. Comp Immunol Microbiol Infect Dis. 2018 Apr;57:22–8.

76.	Bag A, Chattopadhyay RR. Synergistic antibacterial and antibiofilm efficacy of nisin in combination with *p* -coumaric acid against food-borne bacteria *Bacillus cereus* and *Salmonella typhimurium*. Lett Appl Microbiol [Internet]. 2017 Nov;65(5):366–72. Available from: https://academic.oup.com/lambio/article/65/5/366/6704788

77.	Li J, Zhao N, Xu R, Li G, Dong H, Wang B, et al. Deciphering the antibacterial activity and mechanism of p-coumaric acid against Alicyclobacillus acidoterrestris and its application in apple juice. Int J Food Microbiol. 2022 Oct;378:109822.

78.	Chauhan R, Azmi W, Bansal S, Goel G. Multivariate analysis of adaptive response to ferulic acid and *p* ‐coumaric acid after physiological stresses in *Cronobacter sakazakii*. J Appl Microbiol. 2021 Dec 22;131(6):3069–80.

79.	Kim H, Panda PK, Sadeghi K, Lee S, Chung C, Park Y, et al. Facile thermal and hydrolytic conversion of tannic acid: Enhancement of antimicrobial activity and biocompatibility for biomedical applications. Mater Chem Phys. 2022 Jun;285:126141.

80.	Sivasankar C, Jha NK, Ghosh R, Shetty PH. Anti quorum sensing and anti virulence activity of tannic acid and it’s potential to breach resistance in Salmonella enterica Typhi / Paratyphi A clinical isolates. Microb Pathog. 2020 Jan;138:103813.

81.	Jing W, Xiaolan C, Yu C, Feng Q, Haifeng Y. Pharmacological effects and mechanisms of tannic acid. Biomedicine & Pharmacotherapy. 2022 Oct;154:113561.

82.	Aborehab NM, Osama N. Effect of Gallic acid in potentiating chemotherapeutic effect of Paclitaxel in HeLa cervical cancer cells. Cancer Cell Int. 2019;19(1).

83.	Peng W, Luo P, Gui D, Jiang W, Wu H, Zhang J. Enhanced anticancer effect of fabricated gallic acid/CdS on the rGO nanosheets on human glomerular mesangial (IP15) and epithelial proximal (HK2) kidney cell lines - Cytotoxicity investigations. J Photochem Photobiol B. 2018 Jan 1;178:243–8.

84.	Yahya SA, Hassan FA. Cytotoxic Effects Of Gallic Acid In Human Breast Cancer Cell Mda-Mb-231. Journal of Pharmaceutical Negative Results ¦. 13:2022.

85.	Al Balushi N, Hassan SI, Abdullah N, Al Dhahli B, Al Bahlani S, Ahmed I, et al. Addition of Gallic Acid Overcomes Resistance to Cisplatin in Ovarian Cancer Cell Lines. Asian Pacific Journal of Cancer Prevention. 2022;23(8).

86.	Yahya SA, Hassan FA. Cytotoxic Effects Of Gallic Acid In Human Breast Cancer Cell Mda-Mb-231. J Pharm Negat Results [Internet]. 2022 Nov 7 [cited 2023 Jun 13];13:1337–44. Available from: https://pnrjournal.com/index.php/home/article/view/3044

87.	Al Balushi N, Hassan SI, Abdullah N, Al Dhahli B, Al Bahlani S, Ahmed I, et al. Addition of Gallic Acid Overcomes Resistance to Cisplatin in Ovarian Cancer Cell Lines. Asian Pacific Journal of Cancer Prevention [Internet]. 2022 Aug 1 [cited 2023 Jun 13];23(8):2661–9. Available from: https://journal.waocp.org/article_90248.html

88.	Ho IYM, Abdul Aziz A, Mat Junit S. Evaluation of Anti-proliferative Effects of Barringtonia racemosa and Gallic Acid on Caco-2 Cells. Sci Rep. 2020 Jun 19;10(1):9987.

89.	Bai X, Lai T, Zhou T, Li Y, Li X, Zhang H. In vitro antioxidant activities of phenols and oleanolic acid from mango peel and their cytotoxic effect on a549 cell line. Molecules. 2018;23(6).

90.	Bai X, Lai T, Zhou T, Li Y, Li X, Zhang H. In Vitro Antioxidant Activities of Phenols and Oleanolic Acid from Mango Peel and Their Cytotoxic Effect on A549 Cell Line. Molecules 2018, Vol 23, Page 1395 [Internet]. 2018 Jun 8 [cited 2023 Jun 13];23(6):1395. Available from: https://www.mdpi.com/1420-3049/23/6/1395/htm

91.	Sadeghi Ekbatan S, Li XQ, Ghorbani M, Azadi B, Kubow S. Chlorogenic Acid and Its Microbial Metabolites Exert Anti-Proliferative Effects, S-Phase Cell-Cycle Arrest and Apoptosis in Human Colon Cancer Caco-2 Cells. Int J Mol Sci. 2018 Mar 3;19(3):723.

92.	Salzillo A, Ragone A, Spina A, Naviglio S, Sapio L. Chlorogenic Acid Enhances Doxorubicin-Mediated Cytotoxic Effect in Osteosarcoma Cells. Int J Mol Sci. 2021 Aug 10;22(16):8586.

93.	Sapio L, Salzillo A, Illiano M, Ragone A, Spina A, Chiosi E, et al. Chlorogenic acid activates ERK1/2 and inhibits proliferation of osteosarcoma cells. J Cell Physiol. 2020 Apr 10;235(4):3741–52.

94.	Wang X, Liu J, Xie Z, Rao J, Xu G, Huang K, et al. Chlorogenic acid inhibits proliferation and induces apoptosis in A498 human kidney cancer cells via inactivating PI3K/Akt/mTOR signalling pathway. Journal of Pharmacy and Pharmacology. 2019 Jun 7;71(7):1100–9.

95.	Zeng A, Liang X, Zhu S, Liu C, Wang S, Zhang Q, et al. Chlorogenic acid induces apoptosis, inhibits metastasis and improves antitumor immunity in breast cancer via the NF‑κB signaling pathway. Oncol Rep. 2020 Dec 9;45(2):717–27.

96.	Atalay A, Bender O. Evaluation of anti-proliferative and cytotoxic effects of chlorogenic acid on breast cancer cell lines by real-time, label-free and high-throughput screening. Marmara Pharm J. 2018 Apr 6;22(2):173–9.

97.	Iraji A, Firuzi O, Khoshneviszadeh M, Tavakkoli M, Mahdavi M, Nadri H, et al. Multifunctional iminochromene-2H-carboxamide derivatives containing different aminomethylene triazole with BACE1 inhibitory, neuroprotective and metal chelating properties targeting Alzheimer’s disease. Eur J Med Chem. 2017 Dec 1;141:690–702.

98.	Kabała-Dzik A, Rzepecka-Stojko A, Kubina R, Wojtyczka RD, Buszman E, Stojko J. Caffeic Acid Versus Caffeic Acid Phenethyl Ester in the Treatment of Breast Cancer MCF-7 Cells: Migration Rate Inhibition. Integr Cancer Ther. 2018 Dec 24;17(4):1247–59.

99.	Naumowicz M, Kusaczuk M, Zając M, Jabłońska-Trypuć A, Mikłosz A, Gál M, et al. The influence of the pH on the incorporation of caffeic acid into biomimetic membranes and cancer cells. Sci Rep. 2022 Mar 7;12(1):3692.

100.	Secme M, Mutlu D, Elmas L, Arslan S. Assessing effects of caffeic acid on cytotoxicity, apoptosis, invasion, GST enzyme activity, oxidant, antioxidant status and micro-RNA expressions in HCT116 colorectal cancer cells. South African Journal of Botany. 2023 Jun 1;157:19–26.

101.	da Silva LM, Frión-Herrera Y, Bartolomeu AR, Gorgulho CM, Sforcin JM. Mechanisms involved in the cytotoxic action of Brazilian propolis and caffeic acid against HEp-2 cells and modulation of P-glycoprotein activity. Journal of Pharmacy and Pharmacology. 2017 Oct 4;69(11):1625–33.

102.	Caparica R, Rolim Baby A, Almeida T, Guilherme Costa J. In vitro cytotoxicity assessment of ferulic, caffeic and p-coumaric acids on human renal cancer cells. Biomedical and Biopharmaceutical Research Journal. 2020 Mar;17(1):1–12.

103.	Sefidi-Heris Y, Zarei E, Saadat I. Metformin and p-coumaric acid downregulate the expression of hTERT in gastric cancer cell line AGS. Gene Rep. 2023;32.

104.	KARAKURT S, ABUŞOĞLU G, ARITULUK ZC. Comparison of anticarcinogenic properties of Viburnum opulus and its active compound p-coumaric acid on human colorectal carcinoma. TURKISH JOURNAL OF BIOLOGY [Internet]. 2020 Oct 13;44(5):252–63. Available from: https://journals.tubitak.gov.tr/biology/vol44/iss5/4

105.	Zhang Y, Hu M, Liu L, Cheng XL, Cai J, Zhou J, et al. Anticancer effects of Rosmarinic acid in OVCAR-3 ovarian cancer cells are mediated via induction of apoptosis, suppression of cell migration and modulation of lncRNA MALAT-1 expression. J BUON. 2018;23(3):763–8.

106.	Sánchez-Carranza J, Alvarez L, Marquina-Bahena S, Salas-Vidal E, Cuevas V, Jiménez E, et al. Phenolic Compounds Isolated from Caesalpinia coriaria Induce S and G2/M Phase Cell Cycle Arrest Differentially and Trigger Cell Death by Interfering with Microtubule Dynamics in Cancer Cell Lines. Molecules. 2017 Apr 22;22(4):666.

107.	Frisch M; TGW; SHB; SGE; RMA; CJR; SG; BV; MB; PGA. Gaussian 09. Gaussian, Inc.: Wallingford, CT, USA, 2009;

108.	Roy Dennington, Todd A. Keith, John M. Millam. GaussView. Semichem Inc; 2016.

109.	Jabłońska-Trypuć A, Wydro U, Serra-Majem L, Wołejko E, Butarewicz A. The Analysis of Bifenox and Dichlobenil Toxicity in Selected Microorganisms and Human Cancer Cells. Int J Environ Res Public Health. 2019 Oct 27;16(21):4137.

110.	Jabłońska-Trypuć A, Krętowski R, Świderski G, Cechowska-Pasko M, Lewandowski W. Cichoric acid attenuates the toxicity of mesotrione. Effect on in vitro skin cell model. Environ Toxicol Pharmacol. 2020 Jul;77:103375.

111.	Litwinienko G, Ingold KU. Solvent Effects on the Rates and Mechanisms of Reaction of Phenols with Free Radicals. Acc Chem Res. 2007 Mar 1;40(3):222–30.

112.	Vektariene A, Vektaris G, Svoboda J. A theoretical approach to the nucleophilic behavior of benzofused thieno[3,2-b]furans using DFT and HF based reactivity descriptors. Arkivoc. 2009 May 10;2009(7):311–29.

113.	ILO-WHO International Chemical Safety Cards (ICSCs). 2023.

114.	Settimo L, Bellman K, Knegtel RMA. Comparison of the Accuracy of Experimental and Predicted pKa Values of Basic and Acidic Compounds. Pharm Res. 2014 Apr 19;31(4):1082–95.

115.	Alam A, Tamkeen N, Imam N, Farooqui A, Ahmed MM, Tazyeen S, et al. Pharmacokinetic and Molecular Docking Studies of Plant-Derived Natural Compounds to Exploring Potential Anti-Alzheimer Activity. In: In Silico Approach for Sustainable Agriculture. Singapore: Springer Singapore; 2018. p. 217–38.

116.	Hanai T, Hubert J. Hydrophobicity and chromatographic behaviour of aromatic acids found in urine. J Chromatogr A. 1982 Apr;239:527–36.

117.	Linder PW, Voyé A. Potentiometric investigations of the equilibria between caffeic acid and copper(II), zinc(II), iron(II) and hydrogen ions in aqueous solution. Polyhedron. 1987 Jan;6(1):53–60.

118.	Barvinchenko VN, Lipkovskaya NA, Kulik T V., Kartel’ NT. Adsorption of Natural 3-Phenylpropenic Acids on Cerium Dioxide Surface. Colloid Journal. 2019 Jan 6;81(1):1–7.

119.	Naseem S, Laurent AD, Carroll EC, Vengris M, Kumauchi M, Hoff WD, et al. Photo-isomerization upshifts the pKa of the Photoactive Yellow Protein chromophore to contribute to photocycle propagation. J Photochem Photobiol A Chem. 2013 Oct;270:43–52.

120.	Sik B, Kapcsándi V, Székelyhidi R, Hanczné EL, Ajtony Z. Recent Advances in the Analysis of Rosmarinic Acid From Herbs in the *Lamiaceae* Family. Nat Prod Commun. 2019 Jul 15;14(7):1934578X1986421.

121.	Chen W, Shi X, Xu W, McClements DJ, Liu X, Liu F. Effects of different polyphenols on the structure and properties of sodium caseinate films mediated by tyrosinase. J Agric Food Res. 2022 Dec;10:100395.

122.	Adatoz EB, Hendessi S, Ow-Yang CW, Demirel AL. Restructuring of poly(2-ethyl-2-oxazoline)/tannic acid multilayers into fibers. Soft Matter. 2018;14(19):3849–57.

123.	Abrahamse H, Houreld NN. Genetic Aberrations Associated with Photodynamic Therapy in Colorectal Cancer Cells. Int J Mol Sci. 2019 Jul 2;20(13):3254.

**Supporting information**

**S1 Fig. Structures of *p*-coumaric acid (A), caffeic acid (B), rosmarinic acid (C), chlorogenic acid (D), gallic acid (E) and tannic acid (F )**
