# Supplementary material for: Structural characterization and evaluation of antimicrobial and cytotoxic activity of six plant phenolic acids
Source: PLoS One. 2024 Jun 17;19(6):e0299372. doi: 10.1371/journal.pone.0299372 (PMC11182523; doi:10.1371/journal.pone.0299372)
Supplement: Attachment — Submitted filename: Response to Reviewers.docx [file pone.0299372.s008.docx]

**Please find enclosed the answers to reviewers’ comments. Thank you for the comments, suggestions that improved the manuscript.**

**Reviewer #1:** (1) Explanation for varying effects of compounds concentrations on cell viability, why lower concentrations of the some cases significantly increased cell viability, while higher concentrations decreased it?
**Answer**: Thank you for the valuable comment. In both tested lines, decreases in cell viability were observed at higher concentrations of the analyzed compounds. However, in fact, a different response was observed for both tested lines at the lower concentrations analyzed. An increase in proliferation was observed in the Caco-2 line. This may be due to the fact that, although these are lines of colorectal adenocarcinoma, they are different from each other. The DLD-1 line is a cell line that is more tumorigenic and exhibited great chances of resistance and recurrence. However, the Caco-2 line is less tumorigenic. The different response to the applied compounds may result from differences in the structure and functioning of both cell lines. It may also be related to the chemical structure of the analyzed compounds. To explain why low concentrations of some of the tested compounds have a stimulating effect on the Caco-2 line, more thorough and detailed research should be carried out on the mechanisms of action of the tested compounds in both cell lines, which is in our scientific plans for the next experiment. The explanation was added to the manuscript.

(2) The data in Tables 4 and 5 have serious issues in various parts, such as incorrect values.

**Answer**: Table 4 and 5 have been corrected.

**Reviewer #2:** The work aims to do some computational calculation characterizing the chemical structures of selected phenolic acids and presents the results of antimicrobial and cytotoxic studies against two microbial strains and two cell lines.

General comments:
The manuscript is a mix of literature data and experimental results, however the submission is indicated as research work. In the research article the information provided on figure 1 is not suitable. The information provided in tables 4-5 is characteristic for review paper, not for research work. It is not clear what was a purpose of showing it.

**Answer:** Thank you for you valuable comments. Figure 1 as well as the tables 4 and 5 have been moved to the supplementary data. The Introduction has been significantly shortened.

The introduction section is far too long. It seems like a part from the review manuscript. Before the reader goes to the aim of the work is already overwhelmed by the number of mentioned studies.

**Answer:** The Introduction has been significantly shortened.

Also, since so many research was done on the selected phenolic acids, the novelty of this manuscript has to be much more explained and underlined. It is not clear what was a selection criteria to include these phenolic acids.

**Answer:** Thank you for the comment, it helps us to improve the manuscript. The motivation for undertaking the research and the explanation of the selection of compounds are additionally included in the Introduction. “In this study, we will evaluate the antimicrobial activity of these six natural phenolic acids against a bacterial strains living in the digestive system, i.e. *Escherichia coli* and *Lactobacillus rhamnosus* based on 3-(4,5-dimethylthiazol-2-yl)-2,5-diphenyltetrazolium bromide colorimetric assay. Studies of antimicrobial activity aim to determine the suitability of the tested compounds as potential preservatives with antimicrobial properties against selected food pathogens (*E.coli*). Moreover, the studies on the effect of the analysed compounds on *L.* *rhamnosus* were conducted, which are part of the human intestinal microflora to exclude any negative effects on beneficial probiotic bacteria We will also investigate those phenolic acids cytotoxic effects of on two colorectal adenocarcinoma cell lines:Caco-2 and DLD-1 cell lines in order to analyze potential anticancer activity of studied compounds. Additionally, some structural parameters of the phenolic acids such as lipophilicity coefficient (logP) and reactivity of molecules on the basis of parameters calculated in Gaussian 09W program such aslike ionization potential, electron affinity, electronegativity, chemical hardness, chemical softness, dipole moment and electrophilicity index. The six natural phenolic compounds were chosen on purpose. They have proven biological properties such as antioxidant, antimicrobial or chemopreventive evaluated in different model systems of bacteria or cell lines.The chosen ligands are derivatives of two important aromatic acids, i.e. benzoic and cinnamic acids which differ in the presence of vinyl moiety between the aromatic ring and carboxylic group. Moreover they differ in the number of hydroxyl substituents in the ring (i.e. *p*-coumaric, caffeic and gallic acid), some of them are caffeic acid esters (i.e. rosmarinic and chlorogenic acids) or gallic acid ester (i.e. tannic acid). All this structural difference influence on the solubility, lipophilicity and reactivity of molecules. Therefore the following questions may arise: (1) does the number of -OH substituents in the aromatic ring is the main factor that affect the antimicrobial and cytotoxic activity of compounds (toward selected bacteria and cell lines), (2) does the conjugation of caffeic and gallic acid with other molecules influence on the physico-chemical and biological properties of molecules, (c) do the selected experimental (logP) and theoretical (e.g. energy of HOMO or LUMO orbitals) correlates with the biological activity of molecules?”

There is some potential in the selection of structures for the research. They differ by the number of factors, not only by the amount of hydroxyl groups. However this potential was not used for the benefit of this research. The chemical calculation performed are not correlated in the discussion with the observed biological activity. The ionization and lypophilicity influences the penetration of the compound into the cells, hence this information could help to explain the activity or lack of it, however this type of analysis of the generated data was not done. The results and discussion is rather a presentation of results and it lacks the attempt to explain the observations.

**Answer:** Thank you for the comment. The discussion was improved and the structure-activity discussion was in-depth, taking into account theoretical parameters and lipophilicity. The discussion is in the section Results and discussion, Antimicrobial activity, Cytotoxic activity and final remarks in Conclusions.

Other remarks:
LogP and pKa values of studied phenolic acids are available in the PubChem database, so what was a purpose to calculate them?

**Answer:** The table was removed to supplementary. Collecting data in one place shortens the time needed to search it.

Table 4 and 5 are shown before tables 1-3.

**Answer:** It was corrected.

What solvent was used to prepare working concentrations of studied phenolic acids in the antimicrobial test?

**Answer**: The stock solution was prepared by dissolving it in TrisHCl buffer prior analysis.

Table 6 does not show the units of the presented values. Some of the information presented in this table is already available in the literature, eg: <https://doi.org/10.1002/bio.2932>

**Answer:** We agree. The table was removed to supplementary.

**Reviewer #3:** The article is on the structural characterization and evaluation of the antimicrobial and cytotoxic activity of six plant phenolic acids. It is a well-designed article, fictionally. There are good results in the article. It has high citation potential.

This article can be publish with minor revision. But introduction is so long, please write short

**Answer:** The Introduction has been significantly shortened.

**Reviewer #4:** The manuscript is well-written and presents a comprehensive analysis. However, I encountered difficulty in interpreting the figures (bar graph). The figures appear unclear, hindering a clear understanding of the presented data and its significance. It is crucial that the figures are enhanced for better visibility and comprehension.

**Answer:** Thank you for the comments. The figures were corrected.

**Journal Requirements:**

2. We noticed you have some minor occurrence of overlapping text with the following previous publication(s), which needs to be addressed:

- Biological Activity of New Cichoric Acid–Metal Complexes in Bacterial Strains, Yeast-Like Fungi, and Human Cell Cultures In Vitro - <https://doi.org/10.3390/nu12010154>

**Answer**: Thank you for the comments. The experimental part, which is similar in both publications, has been corrected the to get rid of repetitions in the description of the methodology.

3. In your revision ensure you cite all your sources (including your own works), and quote or rephrase any duplicated text outside the methods section. Further consideration is dependent on these concerns being addressed.

**Answer:** Thank you. It was checked.

4. Thank you for stating the following financial disclosure: 
National Science Centre research project number 2018/29/B/NZ9/01997

Please state what role the funders took in the study.  If the funders had no role, please state: "The funders had no role in study design, data collection and analysis, decision to publish, or preparation of the manuscript." 
If this statement is not correct you must amend it as needed. 
Please include this amended Role of Funder statement in your cover letter; we will change the online submission form on your behalf.

**Answer**: It is correct, the funder (National Science Centre) had no role in study design, data collection and analysis, decision to publish, or preparation of the manuscript.

5. Thank you for stating the following in the Acknowledgments Section of your manuscript: 
This research was funded by National Science Centre (NCN), Poland, under the research project number 2018/29/B/NZ9/01997.

We note that you have provided funding information that is not currently declared in your Funding Statement. However, funding information should not appear in the Acknowledgments section or other areas of your manuscript. We will only publish funding information present in the Funding Statement section of the online submission form. 
Please remove any funding-related text from the manuscript and let us know how you would like to update your Funding Statement. Currently, your Funding Statement reads as follows: 
National Science Centre research project number 2018/29/B/NZ9/01997

Please include your amended statements within your cover letter; we will change the online submission form on your behalf.

**Answer**: I understand. The information about funding was deleted from the Acknowledgement and removed to the Funding Statement.

6. In your Data Availability statement, you have not specified where the minimal data set underlying the results described in your manuscript can be found. PLOS defines a study's minimal data set as the underlying data used to reach the conclusions drawn in the manuscript and any additional data required to replicate the reported study findings in their entirety. All PLOS journals require that the minimal data set be made fully available. For more information about our data policy, please see <http://journals.plos.org/plosone/s/data-availability>.

**Answer:** The data that support the findings of this study is openly available in a repository (doi: 10.24427/2018/29/b/nz9/01997_pb; URL: https://bazawiedzy.pb.edu.pl/info/researchdata/BUT1d8312e294574d51aee166b78f760993/).
